# Cnm1 mediates nucleus–mitochondria contact site formation in response to phospholipid levels

**DOI:** 10.1083/jcb.202104100

**Published:** 2021-10-25

**Authors:** Michal Eisenberg-Bord, Naama Zung, Javier Collado, Layla Drwesh, Emma J. Fenech, Amir Fadel, Nili Dezorella, Yury S. Bykov, Doron Rapaport, Ruben Fernandez-Busnadiego, Maya Schuldiner

**Affiliations:** 1 Department of Molecular Genetics, Weizmann Institute of Science, Rehovot, Israel; 2 Institute for Neuropathology, Georg August Universität Göttingen, Göttingen, Germany; 3 Cluster of Excellence “Multiscale Bioimaging: from Molecular Machines to Networks of Excitable Cells,” University of Göttingen, Göttingen, Germany; 4 Interfaculty Institute of Biochemistry, University of Tuebingen, Tuebingen, Germany; 5 Electron Microscopy Unit, Chemical Research Support, Weizmann Institute of Science, Rehovot, Israel

## Abstract

Mitochondrial functions are tightly regulated by nuclear activity, requiring extensive communication between these organelles. One way by which organelles can communicate is through contact sites, areas of close apposition held together by tethering molecules. While many contacts have been characterized in yeast, the contact between the nucleus and mitochondria was not previously identified. Using fluorescence and electron microscopy in *S. cerevisiae*, we demonstrate specific areas of contact between the two organelles. Using a high-throughput screen, we uncover a role for the uncharacterized protein Ybr063c, which we have named Cnm1 (contact nucleus mitochondria 1), as a molecular tether on the nuclear membrane. We show that Cnm1 mediates contact by interacting with Tom70 on mitochondria. Moreover, Cnm1 abundance is regulated by phosphatidylcholine, enabling the coupling of phospholipid homeostasis with contact extent. The discovery of a molecular mechanism that allows mitochondrial crosstalk with the nucleus sets the ground for better understanding of mitochondrial functions in health and disease.

## Teaser

A high-throughput screen uncovered a role for the uncharacterized protein, Ybr063c (Cnm1 [contact nucleus mitochondria 1]), as a molecular tether of the nucleus–mitochondria contact in yeast. Cnm1 on the nucleus mediates contact by interacting with Tom70 on mitochondria. Regulation of Cnm1 abundance by phosphatidylcholine enables coupling of phospholipid levels with contact extent.

## Introduction

During the evolution of eukaryotes, an α-proteobacterium integrated into its archaeal host cell, giving rise to the mitochondrial organelle (Dyall et al., 2004). As mitochondrial genes transferred to the nuclear genome, the response to mitochondrial stress also became nuclear transcribed, and mitochondria number and function had to become coordinated with cellular needs and cell division. This increased dependence on the nucleus required that the two organelles evolve methods of communication. The importance of this communication is evident by how its breakdown contributes to a number of diseases, such as various forms of cancer (Mello et al., 2019; Xia et al., 2019; Yi, 2019), fatty liver disease (Yi, 2019), insulin resistance and obesity (Lee et al., 2015), and physiological conditions such as aging (Mohrin et al., 2015; Reynolds et al., 2020).

Over the years, many aspects of nucleus–mitochondria communication have been intensively studied. Signaling cascades between the organelles were found, dually targeted proteins described, and mitochondrial metabolites required for nuclear function characterized (Eisenberg-Bord and Schuldiner, 2017b; English et al., 2020). However, more direct forms of communication between the two organelles, such as through contact sites, were less explored.

Contact sites are areas where the membranes of two organelles are actively tethered by proteins. Contact sites house unique proteins and lipids and allow direct crosstalk between organelles. The short distance between organelles in these contacts (usually ranging between 10 and 80 nm; Scorrano et al., 2019), enables the rapid, efficient and directional transfer of ions, lipids and metabolites (Eisenberg-Bord et al., 2016; Eisenberg-Bord and Schuldiner, 2017a; Zung and Schuldiner, 2020). While contact sites between multiple pairs of organelles have been demonstrated and investigated in some depth (Shai et al., 2018), the contacts between mitochondria and the nucleus remain elusive. One reason for this gap in our knowledge is that the outer nuclear membrane is continuous with the membrane of the most abundant organelle in the cell, the ER. Since the ER forms extensive contacts with mitochondria, it was hence difficult to distinguish a contact that is unique to the nuclear envelope.

The ER–mitochondria contact site was the first to be described in the 1950s (Bernhard and Rouiller, 1956; Bernhard et al., 1952; Copeland and Dalton, 1959). However, it was not until 2009 that the tethering machinery mediating this contact in *Saccharomyces cerevisiae* (from here on termed yeast) was characterized and named the ER–mitochondria encounter structure (ERMES; Kornmann et al., 2009). This tethering complex is composed of one subunit spanning the mitochondrial membrane (Mdm10), one spanning the ER membrane (Mmm1), and two cytosolic subunits (Mdm34 and Mdm12; Kornmann et al., 2009). The ERMES complex was demonstrated to play a role in the transfer of phospholipids between the ER and mitochondria (Kawano et al., 2018; Kundu and Pasrija, 2020; Endo et al., 2018). In recent years, additional tethering machineries for the ER–mitochondria contact in yeast were discovered (Murley et al., 2015; Elbaz-Alon et al., 2015; Gatta et al., 2015; Lahiri et al., 2014), however whether any of these are required for communication between the nuclear envelope and mitochondria was not determined.

Recently, a contact site between the nucleus and mitochondria was described in human mammary cancer tissue and was then further studied in cell lines (Desai et al., 2020). This contact site was shown to have a role in the retrograde signaling response, occurring between the nucleus and mitochondria, and is facilitated by the cholesterol binding and translocator protein TSPO (Desai et al., 2020). The formation of this contact site was independent of two of the tethering machineries facilitating ER–mitochondria contacts in human cells (VAPB5 and mitofusin 2), suggesting that these contacts are distinct (Desai et al., 2020). However, a TSPO homolog is not found in the yeast proteome. In yeast, it has also recently been suggested that a dedicated contact site between mitochondria and the nucleus exists, since heme, created in mitochondria, bypasses cytosolic pools, and transfers directly into the nucleus (Martinez-Guzman et al., 2020). Hence, it became important to prove that such a contact site exists in yeast as well as uncover its molecular tethers.

Here, we describe a contact between mitochondria and the nuclear periphery (nuclear ER) in yeast that is ERMES independent. Using high-content screens, we find a dedicated tether formed by the previously unstudied nuclear envelope protein Ybr063c (which we name Cnm1 [contact nucleus mitochondria 1]) and uncover its interaction partner on mitochondria, the component of the TOM (translocase of outer membrane [OM]) complex, Tom70. We show that Cnm1 and Tom70 are sufficient for contact site formation and that Cnm1-mediated contact sites are regulated by phosphatidylcholine (PC) metabolism. Our studies pave the way for a more comprehensive understanding of nucleus–mitochondria communication.

## Results

### Mitochondria form ERMES-independent contact sites with the nuclear ER

**Figure 1.**
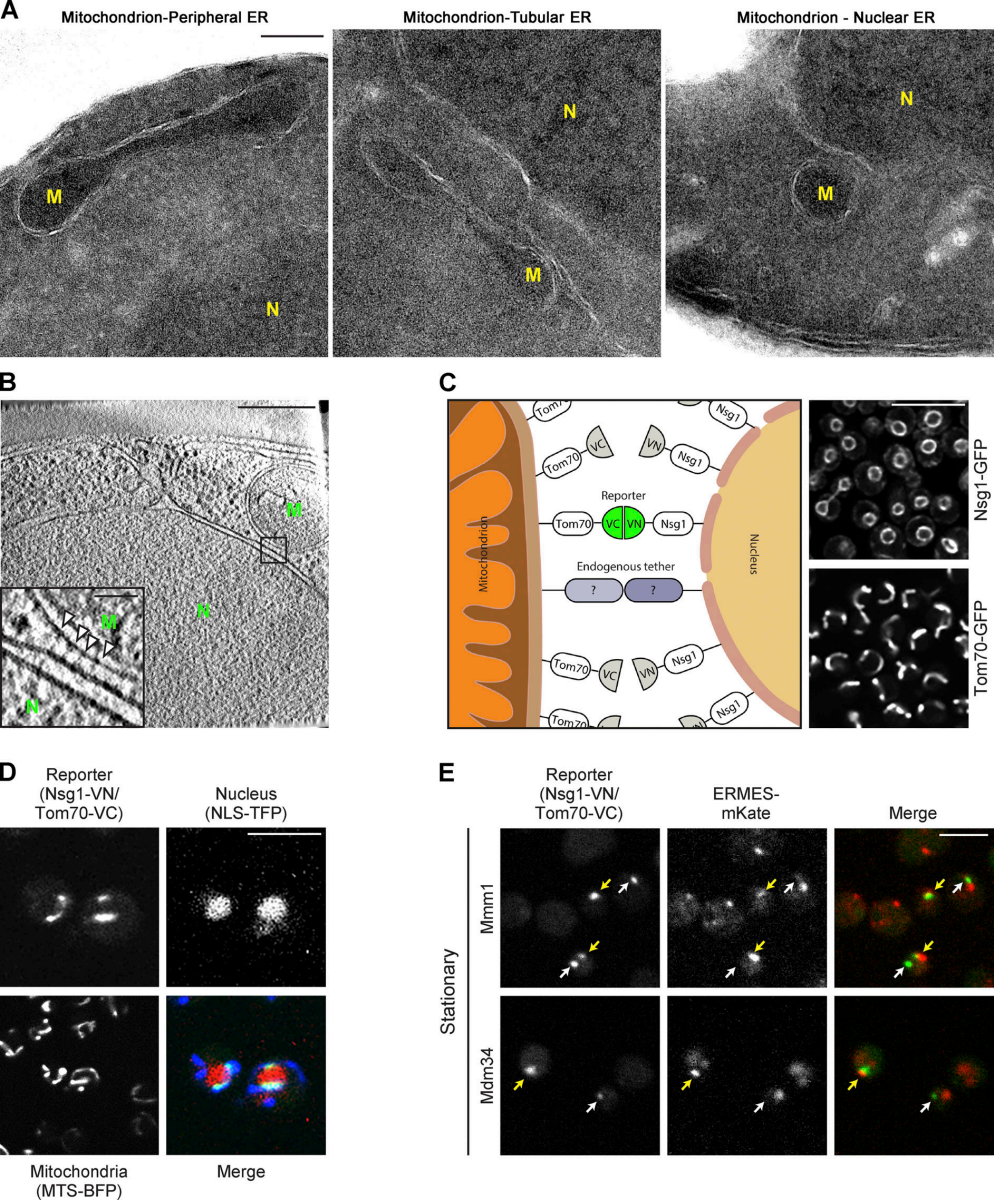
**Mitochondria form contact sites with the nuclear ER that are ERMES independent. (A)** EM images of yeast S288c background demonstrate different mitochondrial contact sites with the various subcompartments of the ER (peripheral, tubular, and nuclear). Each image was differentially adjusted for brightness. M, mitochondrion; N, nucleus. Scale bar, 200 nm. **(B)** Tomograms of yeast (SEY6210.1 background) show the contact sites between the two organelles. M, mitochondrion, N, nucleus, scale bar, 300 nm. Inset: High-density regions that may represent molecular tethers (arrowheads). Scale bar, 50 nm. **(C)** Schematic illustration of a nucleus–mitochondria contact site reporter. The C-terminal part of a Venus protein (VC) was attached to outer mitochondrial membrane protein, Tom70. The N-terminal part of the Venus protein (VN) was attached to the nuclear ER protein Nsg1. These proteins are homogenously distributed on the OM of their respective organelles, as demonstrated by the images when tagged with GFP on their C terminus. Only in cases where the two organelles are in proximity, as in the case of contact sites, the full Venus protein forms and the fluorescent signal is detected. Scale bar, 5 µm. **(D)** The nucleus–mitochondria reporter Nsg1-VN/Tom70-VC correctly identifies proximities between the two organelles. Nuclei are visualized by the red fluorophore (tdTomato) fused to a NLS (NLS-TFP). Mitochondria are visualized by a BFP fused to a mitochondrial targeting sequence (MTS-BFP). The fluorescent signal of the reporter is only localized to areas of proximity between mitochondria and the nucleus. Scale bar, 5 µm. **(E)** Some nucleus–mitochondria contacts are distinct from ERMES-mediated ER–mitochondria contacts. The ERMES subunits Mmm1 and Mdm34 were tagged with mKate and integrated into the nucleus–mitochondria reporter strain. Cells were imaged in stationary phase. Yellow arrows mark areas of colocalization between the ERMES-mKate signal and the reporter, while white arrows mark areas where only the reporter signal is detected (ERMES-independent contacts). Scale bar, 5 µm.

EM images of yeast cells demonstrates three distinct types of contact sites between mitochondria and the ER: those with cortical ER, those with tubular ER, and some with the nuclear ER (which is continuous with the outer nuclear membrane; Fig. 1 A).

**Figure S1.**
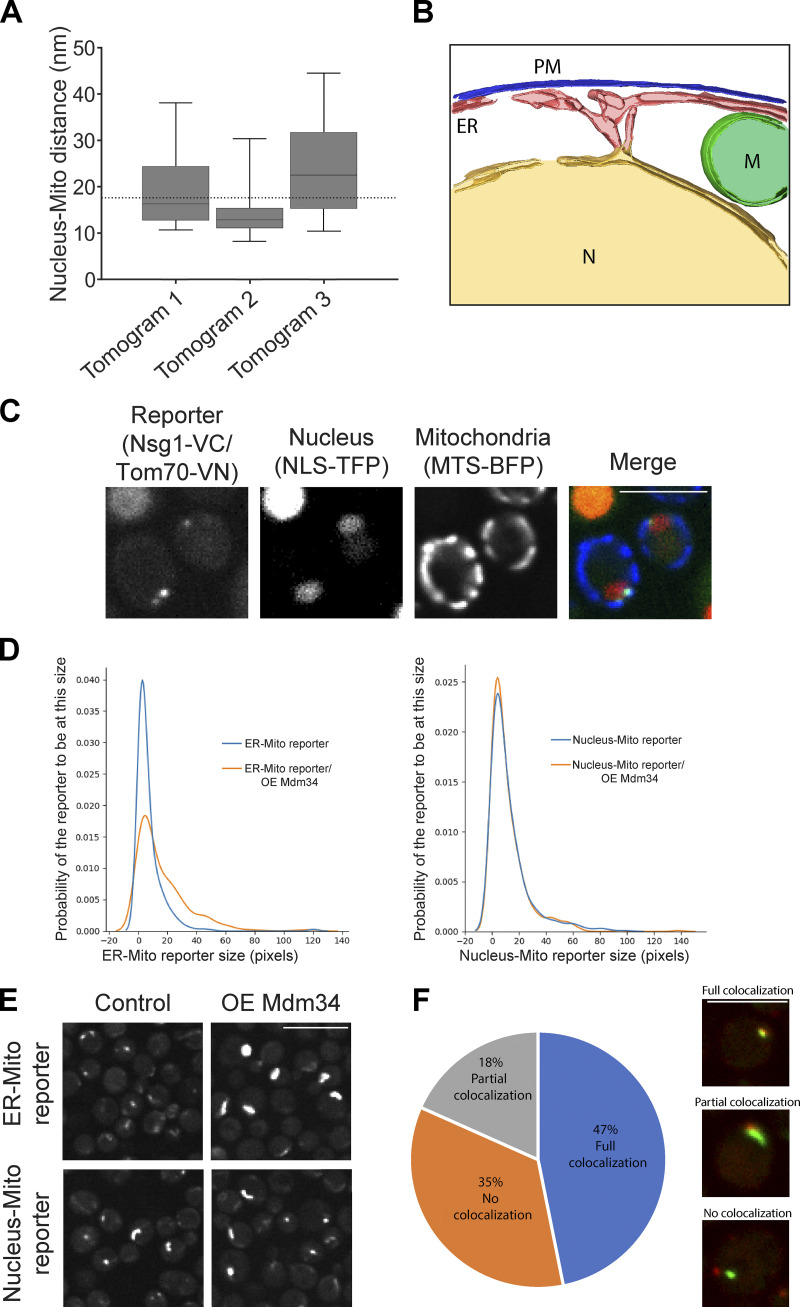
**Overexpression of the ERMES complex does not extend nucleus–mitochondria contacts. (A)** Quantitation of the distances (nanometers) between the nuclear and mitochondrial membranes in a SEY6210.1 strain as determined by three different tomograms. The boxes represent the interquartile range of distance measurements per tomogram (tomogram 1: *n* = 27,743; tomogram 2: *n* = 40,401; tomogram 3: *n* = 9,509); bars mark 0.95 and 0.05 percentiles. The line at the center of the box represents the median. The dotted line represents the mean distance of all three samples. **(B)** 3D segmentation of the nucleus–mitochondria contact in yeast based on the tomogram in Fig. 1 B. M, mitochondrion; N, nucleus; PM, plasma membrane. **(C)** The nucleus–mitochondria reporter Nsg1-VC/Tom70-VN correctly identifies proximities between the two organelles. Nuclei are visualized by the red fluorophore (tdTomato) fused to a nuclear localization signal (NLS-TFP) . Mitochondria are visualized by a BFP fused to a mitochondrial targeting sequence (MTS-BFP). The fluorescent signal of the reporter is only localized to areas of proximity between mitochondria and the nucleus. Scale bar, 5 µm. **(D)** Quantitation of either the nucleus–mitochondria reporter (Nsg1-VN/Tom70-VC) or the ER–mitochondria reporter (Tom70-VN/Pho88-VC) sizes in control strains or those overexpressing Mdm34 (OE Mdm34) N terminally tagged with mCherry. The reporter sizes were determined by the number of pixels of the reporter signal using ScanR Olympus soft imaging solutions version 3.2. While Mdm34 overexpression affects the ER–mitochondria reporter, it does not alter the nucleus–mitochondria one. **(E)** An example of the effect of overexpressing Mdm34 N terminally tagged with mCherry on the background of the two reporters in D. Scale bar, 5 µm. **(F)** Statistical analysis of colocalization between the nucleus–mitochondria contact reporter and the ERMES subunit Mmm1 tagged with mKate on its C terminus. Cells were imaged in stationary phase, and colocalization events were counted manually using a cell counter plugin in ImageJ (Schindelin et al., 2012). Full colocalization was denoted in cases where both punctate signals were completely overlapping (see top image), partial colocalization was designated if the Mmm1 signal only colocalized with a small fraction of the reporter signal (see middle image), whereas no colocalization was scored when the reporter did not overlap any Mmm1 signal whatsoever (see bottom image). Scale bar, 5 µm; *n* = 400.

To corroborate the existence of mitochondria–nuclear ER (from here on called nucleus) contact sites and observe them at higher resolution, we used focused ion beam thinning of vitrified yeast and cryoelectron tomography (Collado and Fernández-Busnadiego, 2017; Collado et al., 2019). The enhanced resolution and sample preservation enabled us to not only measure (Salfer et al., 2020) the average distance between the nucleus and mitochondria in these areas to be ∼20 nm, as would be expected from a bone fide contact site (Scorrano et al., 2019; Fig. S1 A), but also model the contact by 3D segmentation (Salfer et al., 2020; Fig. S1 B). Moreover, we could visualize native protein densities at the contact area that may represent specific tethering molecules underlying the formation of this contact site (Fig. 1 B).

To find potential tethers and resident proteins of the nucleus–mitochondria contact site, we first developed a method to visualize the contact using fluorescence microscopy. We used the split Venus approach for building a contact site reporter in the absence of prior knowledge as to the identity of the molecular tethers (Shai et al., 2018). In short, we attached one part of a split Venus molecule to Tom70, an outer mitochondrial membrane protein, and the second part to Nsg1, a nuclear periphery protein (see scheme in Fig. 1 C). The correct localization of fluorescently labeled variants of both proteins was confirmed by fluorescence microscopy (Fig. 1 C). Only at contact sites, where the two membranes are in proximity, the Venus fragments interact, the full Venus protein is formed, and the resulting fluorescence enables imaging by a fluorescent microscope. Indeed, we observed a clear fluorescent signal suggesting that the nucleus–mitochondria contact site can be imaged by this approach, and this was independent of the Venus fragment appended to either Nsg1 or Tom70 (Figs. 1 D and S1 C).

To verify that the reporters are specific, we imaged them relative to both mitochondria and the nucleus. Indeed, images of these cells verified that in all cases where a signal from the reporter was observed, it occurred at areas of apposition between the nucleus and mitochondria (Figs. 1 D and S1 C), meaning the reporters accurately identify proximity between the two organelles.

To test if our reporter is simply reflecting contact sites facilitated by the well-studied ER–mitochondria contact site machinery, we overexpressed one ERMES subunit, Mdm34, and analyzed its effect on our reporter and on an ER–mitochondria contact reporter as a control. It has been well documented that overexpressing a tether can expand contact extent (Shai et al., 2018). Indeed, we found that overexpression of Mdm34 caused the appearance of cells with increased extent of the ER–mitochondria contact site reporter. However, it did not extend the nucleus–mitochondria contact site reporter (Fig. S1, D and E), suggesting that the reporter is showing an ERMES-independent structure. We then visualized the nuclear–mitochondria contact reporter relative to two ERMES components, Mmm1 and Mdm34. The pattern of colocalization between Mmm1/Mdm34 and the reporter highlighted the existence of two distinct populations. Some reporter signals were in close proximity to Mmm1 (47%) or Mdm34 signals; however, some were only partially colocalized with Mmm1 (18%) or Mdm34, and others were completely distinct from ERMES subunits (35% for Mmm1 signals), demonstrating that nucleus–mitochondria proximity can be ERMES independent (Figs. 1 E and S1 F). These observations suggested that distinct tethering molecules facilitate the contact site between mitochondria and the nucleus.

### High-content screens reveal residents and effectors of the nucleus–mitochondria contact

**Figure 2.**
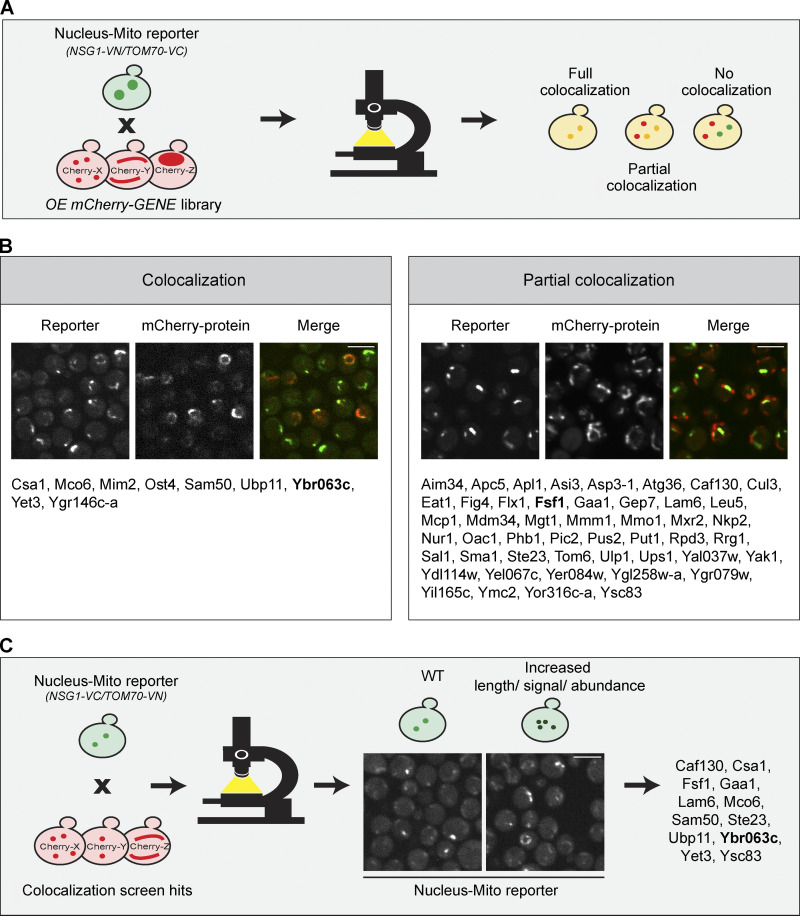
**High-content screens reveal residents and effectors of the nucleus–mitochondria contact. (A) **Illustration of the high-content screen directed at finding resident proteins of the nucleus–mitochondria contact site. The reporter (*NSG1-VN/TOM70-VC*) was integrated into a library of ∼6,000 yeast strains, each harboring an overexpressed and mCherry-tagged version of a different yeast protein. Strains were imaged using automated microscopy, and images were manually examined to identify proteins that colocalize, either fully or partially, with the reporter. **(B)** List of all proteins that either fully (left) or partially (right) colocalized with the reporter, organized by alphabetical order. The proteins shown in the representative images are marked in bold. Scale bars, 5 µm. For a complete list of all proteins and their descriptions, see Table S1. **(C)** Illustration of a screen aimed at identifying effectors of the nucleus–mitochondria contact site. The reporter (*NSG1-VC/TOM70-VN*) was integrated into all 57 hits from the primary screen (shown in B). The effect of their overexpression on the reporter was inspected and 12 hits were found. A representative image shows the protein marked in bold out of the full list of hits. Scale bar, 5 µm.

The first step toward reaching a mechanistic understanding of a contact site is to uncover tethering molecules as well as resident proteins and regulators. To identify such proteins in an unbiased way, we performed a high-content screen using a collection of all yeast proteins tagged with mCherry at their N terminus and overexpressed from a *TEF2* promoter (Weill et al., 2018). Using automated approaches (Cohen and Schuldiner, 2011), we integrated into these strains the reporter of the nucleus–mitochondria contact site (*NSG1-VN/TOM70-VC*). We imaged the resulting ∼6,000 yeast strains using a high-throughput microscopy setup and manually analyzed the images to find proteins that colocalize with the contact site signal (Fig. 2 A). The screen uncovered 48 proteins that partially colocalized with the reporter and 9 proteins that fully colocalized with it (Fig. 2 B; the full list of hits with their description is in Table S1).

To sift out potential tethers from this long list of resident proteins, we searched for those that extended the contact when overexpressed, since it is a known characteristic of a molecular tether (Eisenberg-Bord et al., 2016) We imaged both versions of the reporter on the background of all 57 hits from the primary screen. This secondary screen highlighted 12 hits that both colocalized with the reporter and increased its signal (Fig. 2 C), placing them as potential tethering molecules.

### Ybr063c (Cnm1) has the characteristics of a molecular tether

Out of the 12 candidate tethers uncovered by our screens, the protein that seemed most likely to be a direct tether was Ybr063c, an uncharacterized protein of unknown function. Ybr063c was fully colocalized with the reporter (Fig. 2 B), and its overexpression affected the extent of the reporter signal (Fig. 2 C). Moreover, it was predicted by several algorithms to be an integral membrane protein (Weill et al., 2019), a trait important for creating a tethering force. Finally, it was not previously studied or implicated in ER–mitochondria contacts. Hence, we decided to follow up on this protein.

**Figure S2.**
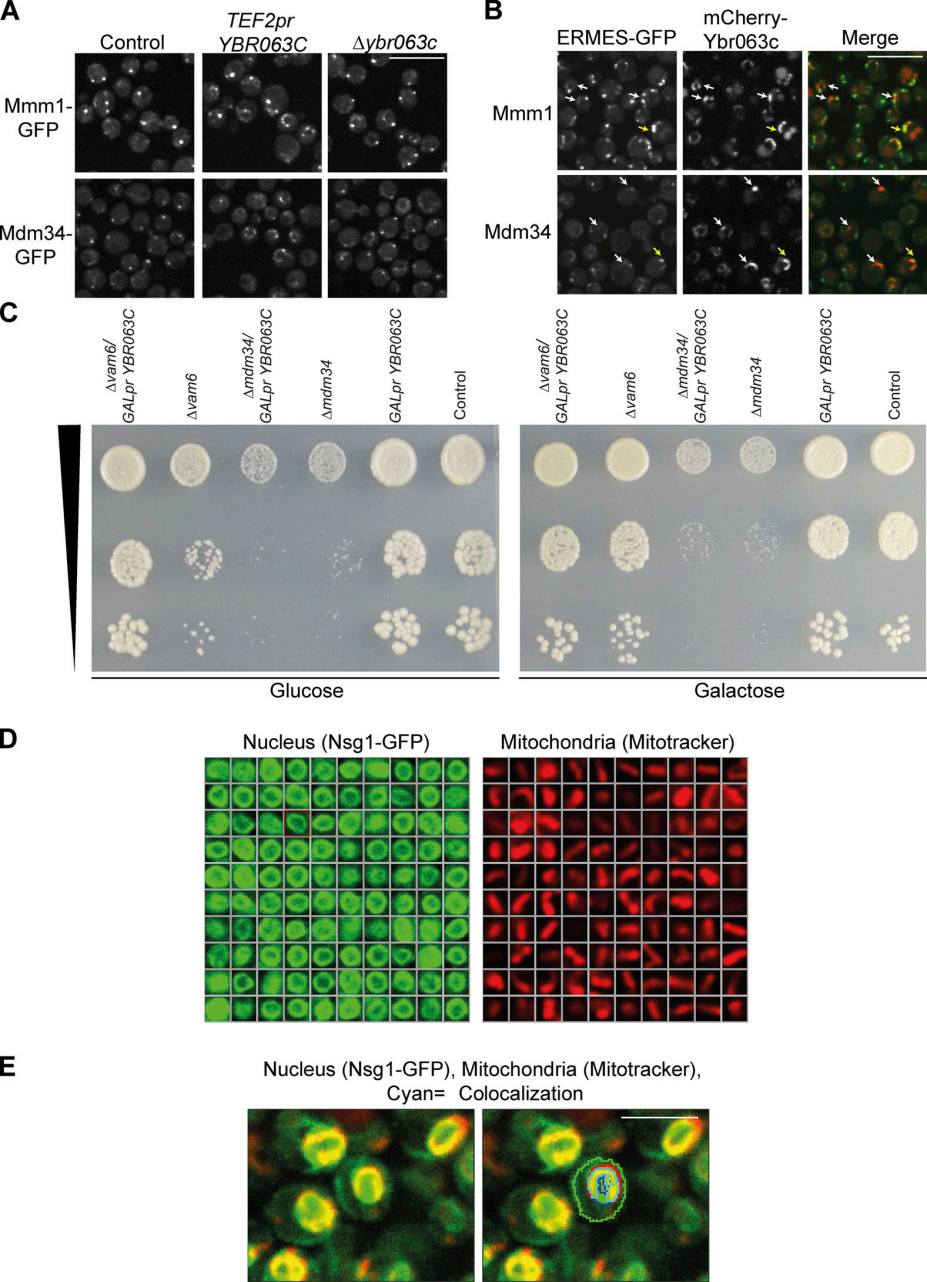
**Ybr063c (Cnm1) does not affect the extent of ERMES-mediated contacts. (A)** ERMES components Mmm1 or Mdm34 were C terminally tagged with GFP on the background of a control strain or strains overexpressing *YBR063C* (*TEF2pr-YBR063C*) or deleted for it *(Δybr063c*). Overexpression of Ybr063c resulted in clustering of ERMES signal to the nuclear ER area but did not change the number or intensity of ERMES puncta. Deleting *ybr063c* had no effect on these proteins. Scale bar, 5 µm. **(B)** Ybr063c can be found in distinct areas from ERMES subunits. Overexpressed Ybr063c was N terminally tagged with mCherry on the background of Mdm34 or Mmm1 C terminally tagged with GFP. The yellow arrows represent areas of proximity between the Ybr063c signal and the ERMES proteins, while the white arrows represent areas of Ybr063c signal that does not colocalize with ERMES. Scale bar, 5 µm. **(C)** A spot dilution assay of strains expressing *ybc063c* under a *GAL* promoter in control strains and strains that harbor deletions in *mdm34* or *vam6*. Repressed expression of *ybr063c* when controlled under the *GALpr* and grown in glucose caused a complete rescue of the growth defect of *Δvam6* in glucose. In contrast, repressing *ybr063c* on the background of *Δmdm34* aggravated the severe growth phenotype of this strain. All strains were grown on both synthetic media with glucose (no expression of Ybr063c) or galactose (Ybr063c is expressed) as a control. **(D)** 100 representative samples of either the nucleus (on the left) or mitochondria (on the right) that were considered in the quantification analysis of Fig. 3 D. The nuclei were marked by Nsg1-GFP, while the mitochondria were dyed using MitoTracker Orange. **(E)** Representation of the overlap analysis between the nucleus and mitochondria by artificial intelligence algorithms (ScanR Olympus soft imaging solutions, version 3.2). Mitochondria segmented in the RFP channel (561 nm) are recognized and marked in red, the nucleus segmented in the GFP channel (488 nm) is recognized and marked in blue, and the overlap between them is recognized and marked with cyan. Scale bar, 5 µm.

To verify that Ybr063c is not simply a part of the ER–mitochondria contact site, we analyzed if its overexpression extends the ERMES-mediated contacts. We found that the extent of Mmm1-GFP or Mdm34-GFP patches is not affected by overexpressing or deleting Ybr063c (Fig. S2 A). Moreover, we could observe areas of Ybr063c expression that did not colocalize with ERMES components and vice versa (Fig. S2 B), supporting the idea that Ybr063c is not directly related to the ERMES complex. In support of Ybr063c acting in an ERMES-independent manner, we found that the combination of *Δmdm34* alongside repressed expression of Ybr063c (growth on glucose when expressed from a galactose-inducible promoter) exacerbated the growth defect of the *Δmdm34* strain alone. In addition, it was shown that loss of both vam6 (that reduces mitochondria–vacuole contacts) and ERMES is synthetic lethal (Elbaz-Alon et al., 2014). Cnm1 repression on the background of the deletion in *vam6* not only did not result in lethality but rather completely rescued the growth defect of the *Δvam6* strain, pointing again to a different function (Fig. S2 C).

**Figure 3.**
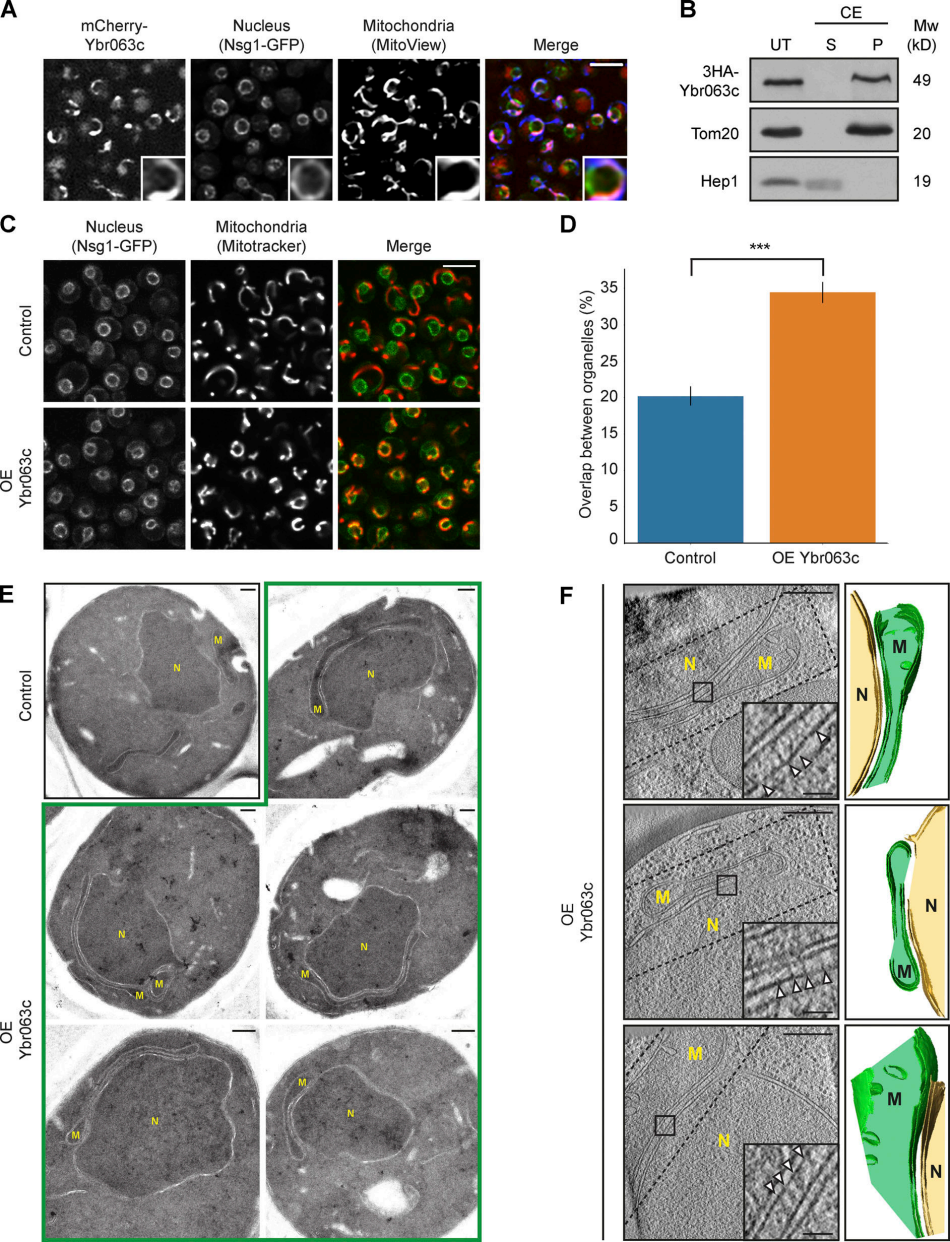
**Ybr063c (Cnm1) has the characteristics of a molecular tether. (A)** Overexpressed mCherry-Ybr063c is localized only to areas of proximity between the nuclear envelope and mitochondria. The nuclear envelope was visualized with Nsg1-GFP and mitochondria by the blue mitochondrial dye (MitoView 405). Insets show an enlarged region of a single nucleus and mitochondria interface with mCherry-Ybr063c signal present where the two organelles connect. Scale bar, 5 µm. **(B)** Ybr063c is a membrane protein embedded in the lipid bilayer. Enriched mitochondrial fractions from cells overexpressing Ybr063c tagged with 3HA on its N terminus were either treated by CE or left untreated (UT). Following this, they were separated into membrane proteins in the pellet (P) or soluble proteins in the supernatant (S). **(C)** Overexpression (OE) of Ybr063c drives clustering of mitochondria around the nucleus. The nuclear membrane was visualized by Nsg1-GFP and mitochondria were stained using a red dye (MitoTracker Orange). Scale bar, 5 µm. **(D)** Quantitation of the proximity between mitochondria and nucleus from C is shown as the percentage of mitochondrial signal that overlaps with the nuclear envelope signal in both a strain that overexpresses Ybr063c (OE Ybr063c) and a control strain. Bars represent standard deviation. *n* = 500; ***, P = 7.24e^−73^. **(E)** EM images of the extended contact sites between nucleus and the mitochondria that are formed by overexpressing Ybr063c (OE Ybr063c, highlighted by a green outline). Scale bars, 200 nm. **(F)** Tomograms of nucleus–mitochondria contacts in yeast overexpressing Ybr063c (OE Ybr063c, left). Scale bars, 300 nm. Insets show high densities that may indicate molecular tethers (arrowheads). Scale bars, 50 nm. 3D segmentations of the contact site area seen in the tomograms (right). The nucleus membrane is marked in yellow, and the mitochondrial membrane is marked in green. Dashed lines on tomograms indicate the area that is seen in the 3D segmentation. M, mitochondrion; N, nucleus.

One of the main characteristics of a molecular tether is its enrichment at the contact site (Eisenberg-Bord et al., 2016). We therefore visualized Ybr063c N terminally tagged with mCherry relative to both the nucleus and mitochondria. Indeed, mCherry-Ybr063c was located at discrete regions on the nuclear envelope that were in contact with mitochondria (Fig. 3 A).

Protein tethers are often integral membrane proteins, enabling them to provide a direct link between the membranes. While some prediction algorithms predicted one or two membrane-spanning domains, others predicted Ybr063c to be a soluble protein (Weill et al., 2019). To test whether Ybr063c is an integral membrane protein, we performed carbonate extraction (CE) on Ybr063c tagged with a small tag (3HA) on its N terminus. CE dissociates peripheral proteins from membranes but cannot extract membrane-embedded polypeptides from the bilayer. Similarly to the mitochondrial OM protein Tom20 and in contrast to the mitochondrial matrix protein Hep1, 3HA-Ybr063c remained in the membrane fraction following this treatment, clearly indicating that it is embedded in the lipid bilayer (Fig. 3 B).

To assay if Ybr063c is sufficient for bringing together the two membranes, we imaged strains overexpressing untagged Ybr063c and monitored the association between mitochondria and the nucleus in the absence of the reporter. Overexpression of Ybr063c under the strong *TEF2* promoter had a striking effect on mitochondrial distribution in the cell, causing clustering of mitochondria around the perinuclear region (Fig. 3 C). Quantification of this proximity showed a nearly twofold increase in proximity between the two organelles (Fig. S2, D and E; and Fig. 3 D). Time-lapse analysis of Ybr063c induction (from a galactose inducible promoter) suggest that this increased proximity is caused by adherence of mitochondria to the nucleus after a random contact between the two organelles has occurred (Video 1).

**Video 1.** **Activating Ybr063c expression from a *GAL* promoter results in mitochondrial adherence to the nucleus when the two organelles come into proximity.** The nucleus is marked by Nsg1-GFP and mitochondria were marked in red using MitoTracker Orange.

Imaging the strains overexpressing Ybr063c by EM demonstrated that these proximities were indeed bona fide contact sites (Fig. 3 E). These results were corroborated by cryoelectron tomography, where 3D segmentation showed abundant nucleus–mitochondria contacts in the strain overexpressing Ybr063c (Fig. 3 F).

Since Ybr063c has the molecular characteristics of a tether affecting extensively the nuclear–mitochondria contact site, we named it Cnm1 for Contact Nucleus Mitochondria 1.

### Identifying factors involved in Cnm1-induced contact sites

**Figure 4.**
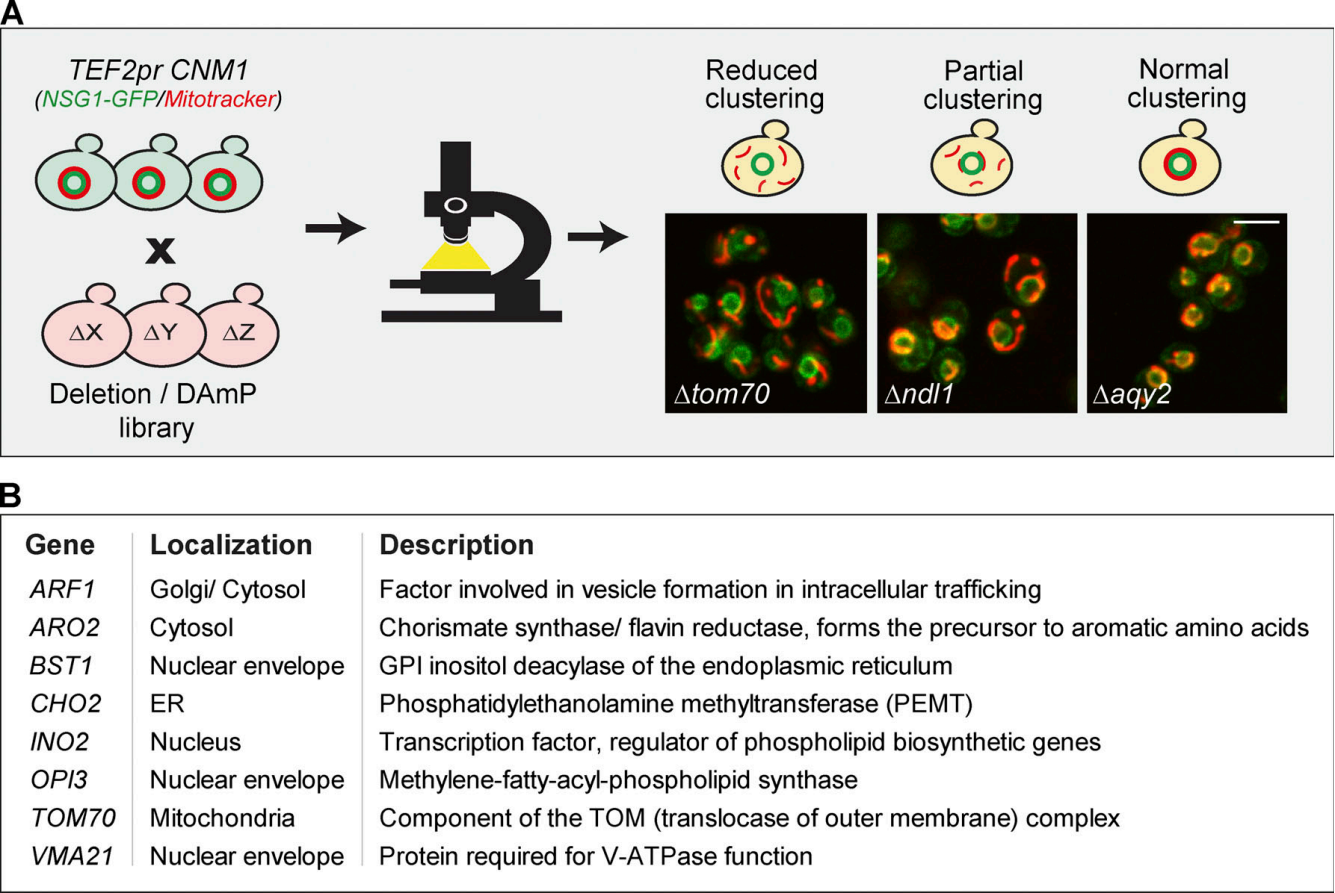
**Identifying factors that are involved in Cnm1-induced contact sites. (A)** A schematic representation of the systematic screen to find modulators of Cnm1 overexpression. Cnm1, overexpressed under the strong *TEF2* promoter, and the nuclear envelope protein Nsg1, tagged with GFP on its C terminus, were integrated into the deletion/hypomorphic allele library. In this library, each colony harbors a loss-of-function mutant in each of the ∼6,000 yeast genes. Prior to imaging, cells were stained with a red mitochondrial dye (MitoTracker Orange). The genes that when mutated resulted in partial or reduced mitochondrial clustering around the nucleus were considered as hits. Representative images of the mutants labeled in white are shown. Scale bar, 5 µm. **(B)** A table of all deleted genes that caused reduced mitochondrial clustering on the background of Cnm1 overexpression arranged by alphabetical order. GPI, glycosylphosphatidylinositol. Protein localization and description are presented in the middle and right columns, respectively. For a full list of the mutant genes that resulted in partial clustering, see Table S2.

**Figure S3.**
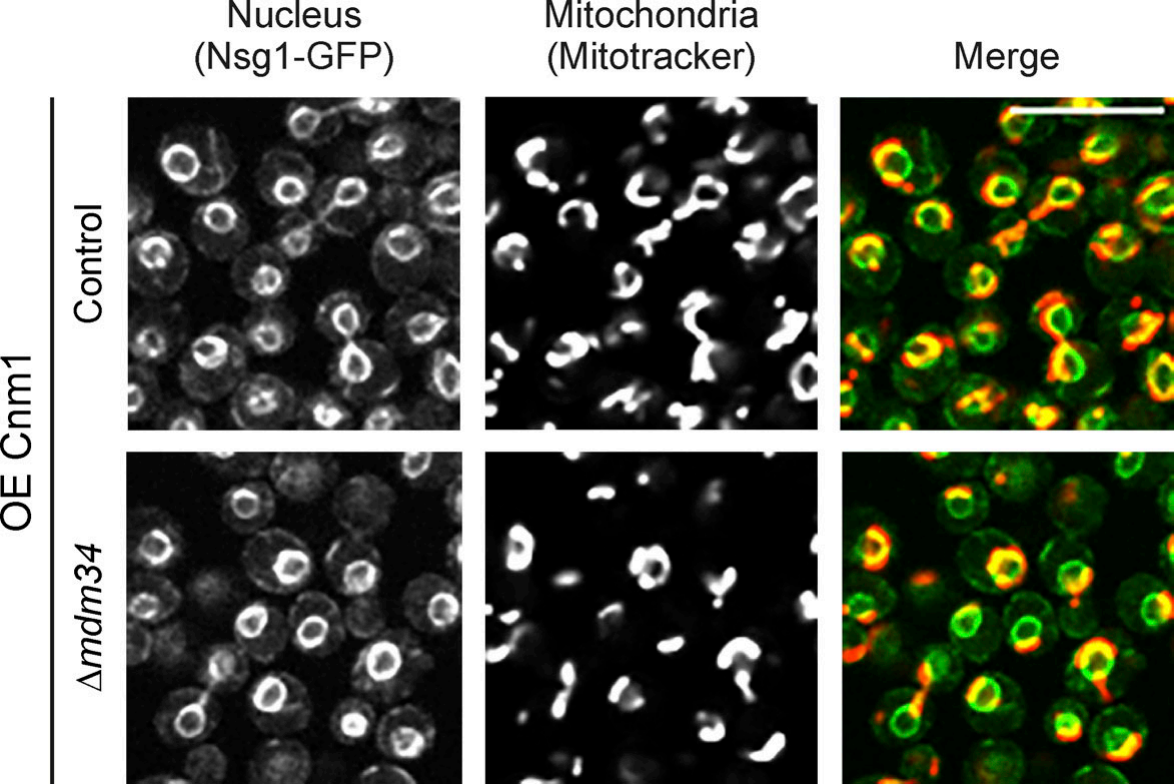
**Cnm1-mediated clustering of mitochondria around the nucleus is ERMES independent. **A strain overexpressing Cnm1 (OE Cnm1) and deleted for the ERMES subunit *mdm34* shows no difference in mitochondrial clustering around the nucleus. The nucleus was visualized with Nsg1-GFP and mitochondria with MitoTracker Orange. Scale bar, 5 µm.

To gain insight on the mechanism of Cnm1-mediated tethering, we set out to find proteins that are required for its ability to promote clustering of mitochondria around the nucleus when overexpressed. We assumed that the deletion of such a gene, which is involved in the clustering of mitochondria around the nucleus, would result in the reversion of this phenotype and less clustering. To search for such a reversion, we integrated overexpressed *CNM1* and a nuclear marker into a collection of mutants in every yeast gene (∼5,000 knockouts of nonessential genes; Deletion library, Giaever et al., 2002; and ∼1,000 hypomorphic alleles of essential ones; Decreased abundance by mRNA perturbation (DAmP) library, Breslow et al., 2008). Next, we performed a high-content microscopy screen on all strains and searched for those that showed less clustering of mitochondria around the nucleus (Fig. 4 A). While 60 genes affected this phenotype to some extent (Table S2), only seven deletions (verified by both check-PCR and remaking the strains to confirm the phenotype; data not shown) completely abolished the effect of *CNM1* overexpression (Fig. 4 B). In support of Cnm1 mediating ERMES-independent contacts, none of the ERMES mutants came up in the screen. and deletion of *mdm34* (verified by check-PCR; data not shown) did not alter the clustering phenotype of *CNM1* overexpression (Fig. S3).

### Cnm1-mediated contact sites are regulated by PC

**Figure 5.**
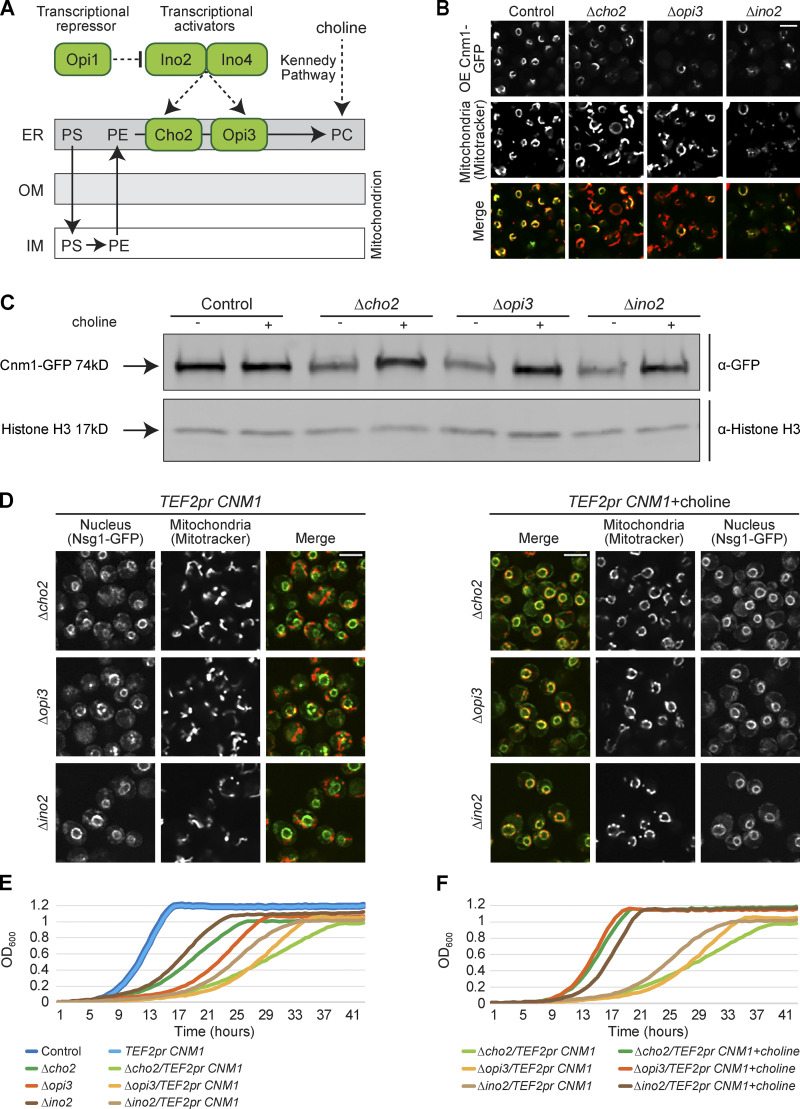
**Cnm1-mediated contact sites are affected by PC metabolism.** **(A)** Schematic illustration of the biosynthesis pathway of PC. PS formed in the ER is transferred to mitochondria to generate PE, which is then transferred back to the ER for the formation of PC by Cho2 and Opi3. Ino2 and Ino4 are the transcriptional activators of both Cho2 and Opi3. Opi1 is a negative regulator of the pathway. PC molecules can also be synthesized through the Kennedy pathway when exogenous choline is present. IM, inner membrane. **(B)** Deletion of PC biosynthesis–related genes reduced Cnm1 signal levels. Overexpressed (OE) Cnm1 was tagged with GFP on its C terminus and mitochondria were stained using MitoTracker Orange. Scale bar, 5 µm. **(C)** Reduced levels of Cnm1-GFP (expressed from a strong constitutive promoter) in strains harboring a deletion of *cho2*, *opi3*, or *ino2* can be rescued by addition of choline. Western blot analysis of four different strains without or with 5mM choline supplementation. Immunoblotting was performed with antibodies against GFP and Histone H3 as a loading control. **(D)** Cnm1 mediated mitochondrial clustering around the nucleus is dependent on choline levels. Cells overexpressing Cnm1 under the *TEF2* promoter and harboring deletion of *cho2*, *opi3*, or *ino2* were grown to mid-logarithmic phase in synthetic minimal medium without or with 5mM choline. The nucleus is visualized by Nsg1-GFP and mitochondria by MitoTracker Orange staining. Scale bar, 5 µm. **(E)** Overexpression of Cnm1 using the *TEF2* promoter in strains deleted for proteins involved in PC biosynthesis resulted in a reduced growth rate. Strains were grown overnight in synthetic minimal medium, back diluted to OD_600_∼0.05 and monitored for growth over 48 h. **(F)** Choline buffered the growth defect of overexpressing Cnm1 in strains deleted for genes involved in PC biosynthesis. Strains were grown overnight in synthetic minimal medium, back diluted to OD_600_∼0.05 and monitored for growth over 48 h with or without 5mM choline supplementation.

Out of the seven hits that most affected the capacity of Cnm1 to cause mitochondrial clustering around the nucleus, we found deletions in three genes (*CHO2*, *OPI3*, and *INO2*) whose protein products are all components of the PC biosynthesis pathway. To produce PC, phosphatidylserine (PS) is converted to phosphatidylethanolamine (PE) mainly in the inner membrane of mitochondria by Psd1, and then PE is transferred back to the ER membrane (Carman and Han, 2011). Both Cho2 and Opi3 are methyltransferases located on the ER membrane, where they convert PE to PC in two enzymatic steps (Fig. 5 A). The PC produced on ER membranes must then be transferred back to mitochondria, where it constitutes 44% of membrane lipids (Sperka-Gottlieb et al., 1988). Ino2 is a transcriptional activator of *CHO2* and *OPI3* genes (Carman and Han, 2011). Identifying three genes of the PC pathway as modulators of Cnm1 activity suggested a connection between PC and the nuclear–mitochondria contact.

Visualizing Cnm1-GFP under regulation of a constitutive promoter and on the background of a deletion of each of the three PC biosynthesis regulators showed a reduction in intensity compared with control. Moreover, we noticed that cells that retained Cnm1 expression still had increased proximity between mitochondria and the nucleus, whereas cells with reduced Cnm1 abundance displayed diminished clustering (Fig. 5 B). Thus, Cho2, Opi3, and Ino2 might affect the capacity to extend the contact by regulating Cnm1 levels.

**Figure S4.**
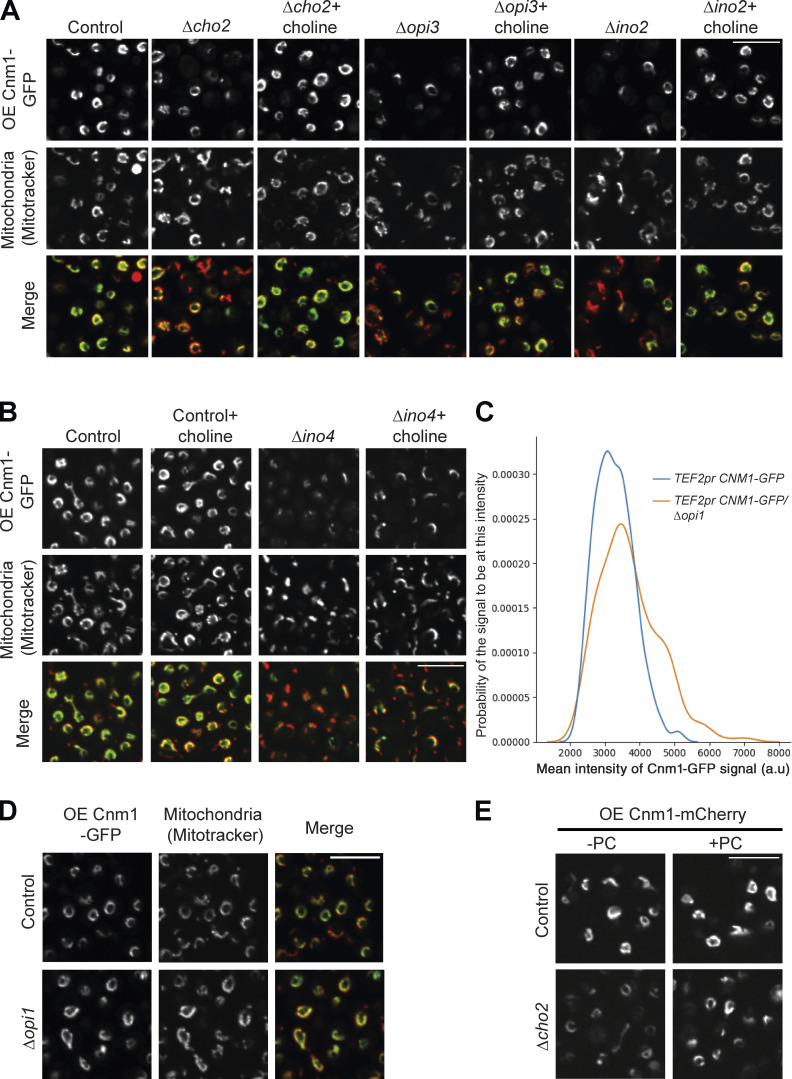
**Choline supplementation rescues reduced Cnm1-GFP levels in strains lacking genes related to PC biosynthesis. (A)** Cells overexpressing Cnm1-GFP (OE Cnm1-GFP) on the background of deletions in *cho2*, *opi3*, and *ino2* were grown to mid-logarithmic phase in synthetic minimal medium and imaged with or without 5 mM choline. Mitochondria were dyed using MitoTracker Orange. Scale bar, 5 µm. **(B)** Cells lacking *ino4* and overexpressing (OE) Cnm1-GFP were grown to mid-logarithmic phase in synthetic minimal medium and imaged with or without 5 mM choline supplementation. Mitochondria were stained using MitoTracker Orange. Scale bar, 5 µm. **(C)** Quantitation of the overexpressed (by *TEF2pr*) Cnm1-GFP signal brightness in either control or *Δopi1* strains, determined by the mean intensity level of the 488-nm excitation wavelength using ScanR Olympus soft imaging solutions, version 3.2. While the mean intensity was maintained in most control cells, deletion of *opi1* resulted in a higher probability of having cells with stronger Cnm1-GFP signal. a.u., arbitrary units. **(D)** An example of the strains quantified in C. Overexpression of Cnm1 tagged with GFP (OE Cnm1-GFP) on its C terminus on the background of *opi1* deletion showed enhanced GFP signal intensity in some of the cells compared with control. Mitochondria were dyed using MitoTracker Orange. Scale bar, 5 µm. **(E)** Cells overexpressing (OE) Cnm1 and C terminally tagged with mCherry on the background of *Δcho2* strain were grown to mid-logarithmic phase in synthetic minimal medium and imaged with or without supplementation of 1 mM PC. Scale bar, 5 µm.

Cho2/Opi3/Ino2 might regulate Cnm1 abundance directly or indirectly through their effect on PC levels. To discriminate between these possibilities, we took advantage of the fact that in yeast, there is a Cho2/Opi3-independent pathway to synthesize PC, the Kennedy pathway. The Kennedy pathway uses externally added choline to conjugate Cytidine 5'-diphosphocholine (CDP-choline) directly to the headgroup of diacylglycerol (Fig. 5 A; Atkinson et al., 1980). Indeed, it was shown that simply adding choline to yeast medium is enough to increase PC levels significantly (Atkinson et al., 1980). Therefore, we assayed whether addition of choline to the growth medium will rescue the levels of Cnm1-GFP in strains lacking Cho2, Opi3, or Ino2. Imaging of these strains shows that this is indeed the case (Fig. S4 A), and this was verified by Western blot analysis (Fig. 5 C).

**Figure S5.**
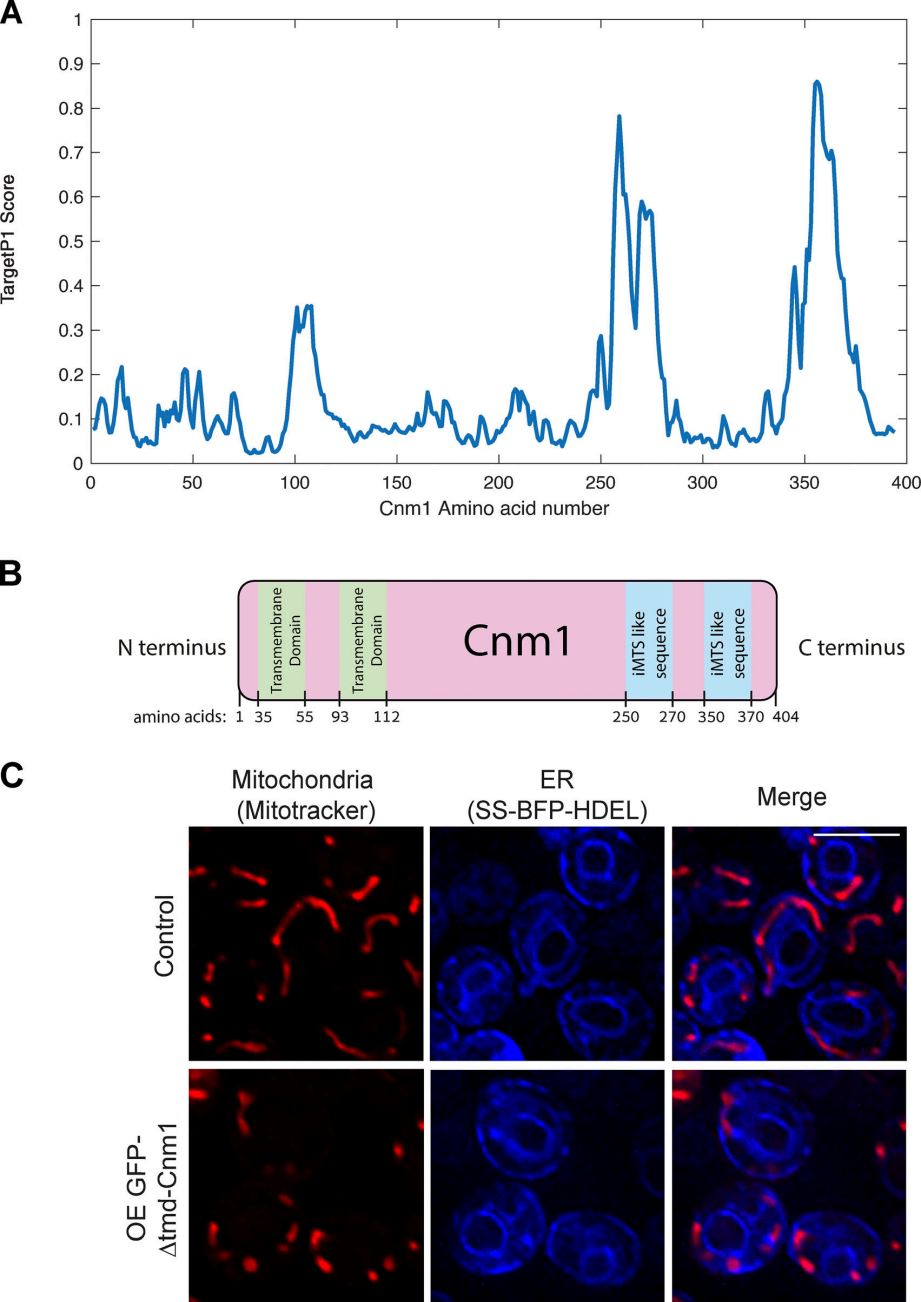
**Domain architecture of Cnm1 and the effect of losing its TMD on mitochondrial morphology.** **(A)** Prediction of an internal mitochondrial targeting signal–like (iMTS-L) sequence in Cnm1 calculated as described before (Boos et al., 2019). A peak with the highest TargetP1 scores can be found around amino acids 350–370 of the nuclear protein Cnm1, suggesting the presence of an iMTS-L sequence in this region. Since iMTS-Ls have been shown to directly bind Tom70, this highlights this region as a potential binding interface of Cnm1 with Tom70 on the mitochondrial membrane. **(B)** An illustrated model of Cnm1 protein containing the localization of its two predicted transmembrane domains and the predicted iMTS-like signals. **(C)** Overexpression of the soluble Cnm1 (*Δ*1–112 aa) tagged with GFP on its N terminus (OE GFP-Δtmd-Cnm1) has a dramatic effect on mitochondrial morphology. The ER is marked by a BFP with a signal sequence and an ER retention signal (SS-BFP-HDEL). Mitochondria were dyed with MitoTracker Orange. Scale bar, 5 µm.

To support this phenotype being a result of PC levels, we assayed the effect of several additional members of this metabolic pathway. We deleted *INO4* encoding for a complex member of Ino2 that is required for its activity as a transcription factor (Carman and Han, 2011; Fig. S4 B), and found that it too reduced Cnm1 abundance and that this phenotype was reversed by addition of choline. Inversely, we deleted *OPI1*, which encodes for a transcriptional repressor that binds the Ino2–Ino4 complex and thus prevents expression of either *CHO2* or *OPI3* (Carman and Han, 2011). As expected, we could quantify more cells with higher intensity of the Cnm1-GFP signal compared with control (Fig. S5, C and D).

We could also rescue Cnm1 levels by supplementation directly with PC, demonstrating that the effect of choline addition was through its integration into PC (Fig. S4 E). Importantly, choline-induced rescue of Cnm1 levels on these backgrounds also restored the contacts between the nucleus and mitochondria (Fig. 5 D). Taken together, these results indicate that PC levels regulate Cnm1 abundance, thus affecting mitochondrial clustering around the nucleus.

Having a tool in hand to pick apart the effect of Cnm1 overexpression, contact site expansion, and PC levels, we turned to assay the effect of these factors on yeast growth. Growth assays demonstrated that inducing clustering of mitochondria around the nucleus by overexpressing Cnm1 does not have an adverse effect on cell growth (Fig. 5 E). Deletion of *cho2*, *opi3*, and *ino2* (in the absence of exogenous choline) reduced growth rate, as would be expected from a diminished capacity to biosynthesize a central phospholipid. Surprisingly, overexpressed Cnm1 exacerbated the adverse effect on growth rate displayed by the strains with reduced PC biosynthesis (*Δcho2*, *Δopi3*, and *Δino2*; Fig. 5 E). The phenotype of overexpressed Cnm1 upon deletions of the three PC biosynthetic genes was also rescued by the addition of choline (Fig. 5 F), suggesting that during conditions of low PC abundance, increasing nucleus–mitochondrial contact is deleterious to cells, potentially due to the shunting of too much PC into mitochondrial membranes.

### Cnm1-mediated contact sites require Tom70

**Figure 6.**
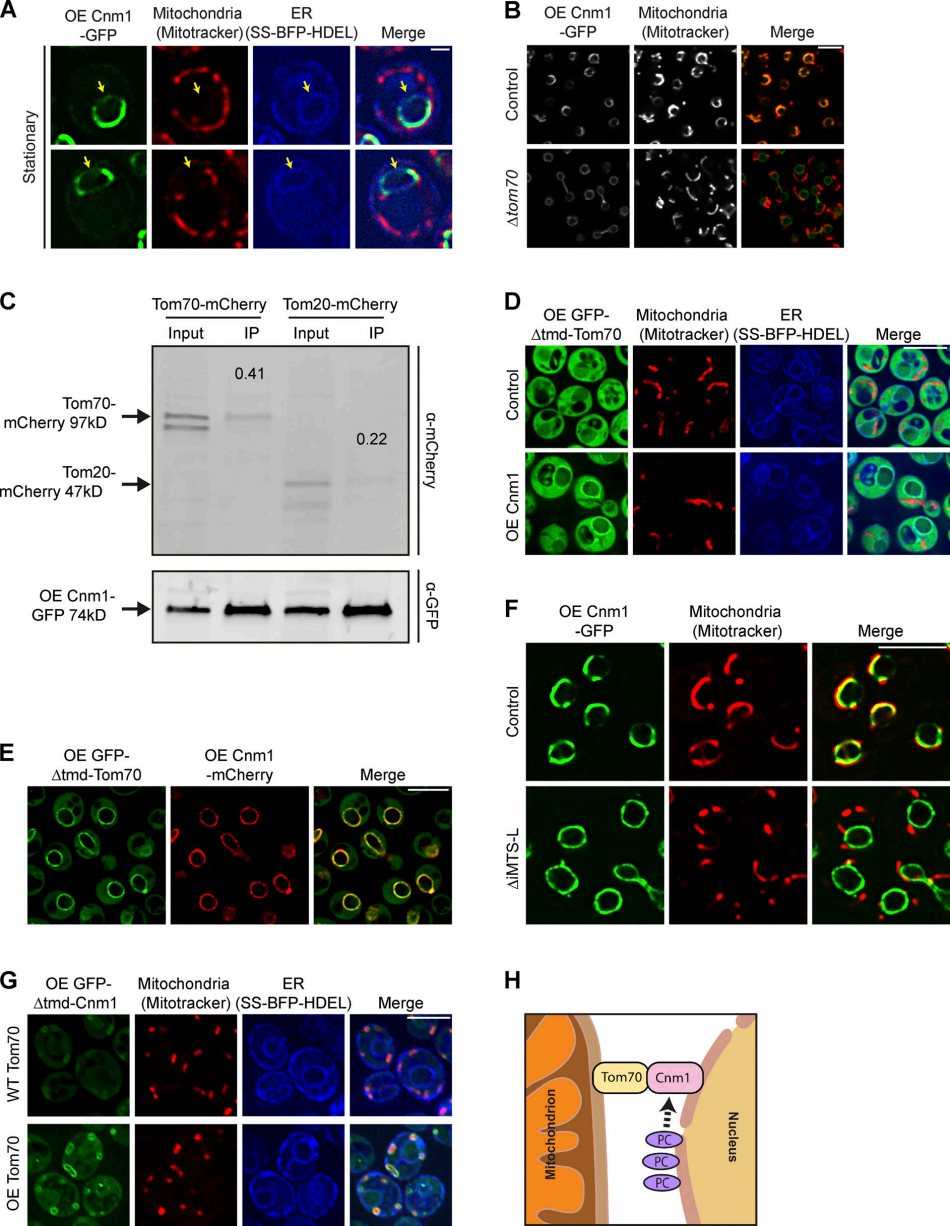
**Cnm1-mediated contact sites require Tom70. (A)** Cnm1 is an outer nuclear membrane protein. A strain overexpressing Cnm1-GFP (OE Cnm1-GFP) during stationary phase shows areas where Cnm1 is not localized to mitochondria (stained by MitoTracker Orange) but does colocalize with the outer nuclear membrane (nuclear ER) visualized using a BFP with a signal sequence and an ER retention signal (SS-BFP-HDEL). Arrows mark areas where Cnm1-GFP signals colocalize with the nuclear ER signal, but not with the mitochondrial signal. Scale bar, 1 µm. **(B)** Loss of *tom70* results in Cnm1 redistributing uniformly around the nucleus. Shown are strains overexpressing (OE) Cnm1-GFP on the background of *Δtom70* or control cells, imaged in mid-logarithmic phase using MitoTracker Orange for mitochondrial staining. Scale bar, 5 µm. **(C)** Tom70 physically interacts with Cnm1. Pull-down of overexpressed Cnm1 tagged with GFP on its C terminus in strains expressing either Tom70 or Tom20 tagged with mCherry on their C termini. Coimmunoprecipitation (co-IP) samples were analyzed by Western blotting and probed with antibodies against GFP and mCherry. Input (10% of total immunoprecipitates) is shown. The number above each immunoprecipitation band represents the enrichment of the protein. **(D)** Overexpression (OE) of Cnm1 results in the accumulation of soluble GFP-Tom70 around the nuclear membrane. Overexpressed Tom70 whose TMD (1–38 aa) has been truncated and is tagged with GFP on its N terminus (OE GFP-Δtmd-Tom70) shows cytosolic distribution in control cells. Overexpression of Cnm1 concentrates the soluble Tom70 around the nuclear membrane marked by a BFP with a signal sequence and an ER retention signal (SS-BFP-HDEL). Mitochondria were dyed with MitoTracker Orange. Control and overexpressed Cnm1 strains are adjusted to different intensities. Scale bar, 5 µm. **(E)** Overexpressed (OE) GFP-Δtmd-Tom70 is fully colocalized with overexpressed Cnm1-mCherry on the nuclear periphery. Scale bar, 5 µm. **(F)** Deletion of the predicted iMTS-L sequence of Cnm1 (350–404 aa) abrogates mitochondrial clustering around the nucleus and results in redistribution of Cnm1 over the entire nuclear membrane. Cnm1-GFP (full length or mutant) were expressed under a *TEF2* promoter. Mitochondria are dyed with MitoTracker Orange. Scale bar, 5 µm. **(G)** Soluble Cnm1 decorates the mitochondrial OM. Overexpressed Cnm1 truncated at its N terminus by fusion of a GFP molecule to remove its predicted TMD (1–112 aa; OE GFP-Δtmd-Cnm1) was expressed in either WT Tom70 cells or cells overexpressing Tom70 (OE Tom70) under the *NOP1* promoter. The nuclear envelope is visualized by a BFP with a signal sequence and an ER retention signal (SS-BFP-HDEL; SS-BFP-HDEL in WT Tom70 and OE Tom70 strains is adjusted to different intensities). Mitochondria are marked by MitoTracker Orange. In control cells, GFP-Δtmd-Cnm1 shows cytosolic distribution as well as enrichment around the mitochondrial periphery and no nuclear periphery staining. Overexpression of Tom70 causes an even brighter signal to accumulate around mitochondria, suggesting that its levels are restrictive to Cnm1 recruitment to mitochondrial surfaces. Scale bar, 5 µm. **(H)** Schematic working model on Cnm1 activity in mediating nucleus–mitochondria contacts. PC levels regulate Cnm1 abundance in the cell. Cnm1 on the nuclear ER membrane interacts with Tom70 on the mitochondrial membrane.

Our results thus far show that Cnm1 has the capacity to form contacts when overexpressed and that its reduced abundance caused reduced contact formation. Next, we wanted to explore the molecular mechanism of tethering between the nucleus and mitochondria. We first ascertained in which of the two organelles Cnm1 resides. We imaged Cnm1-GFP relative to mitochondria and the nucleus during stationary phase. In this condition, there is reduced clustering of mitochondria around the nucleus even when Cnm1 is overexpressed, and this enables better discrimination between the organelles. In this condition, Cnm1 was still highly enriched in the contact area but could also clearly be detected in areas of the nuclear ER that were not adjacent to mitochondria (Fig. 6 A). Since Cnm1 is an integral membrane protein (Fig. 3 B), this places it as a new nuclear membrane resident.

Finding that Cnm1 is a nuclear protein encouraged us to identify its mitochondrial tethering partner. Of the seven hits that dramatically alleviated Cnm1-mediated clustering (Fig. 4, A and B) only one was mitochondrial, Tom70. Moreover, Tom70 was previously shown to act as a tethering partner to Lam6 in the ER–mitochondria contact site (Elbaz-Alon et al., 2015; Murley et al., 2015). Interestingly, Lam6 also came up in our initial screen for proteins affecting the nucleus–mitochondria contact (Fig. 2 A).

To investigate whether Tom70 could be a tethering partner for Cnm1, we imaged cells which overexpressed Cnm1-GFP in a *Δtom70* background. Indeed, a dramatic effect on Cnm1 localization was observed and Cnm1-GFP could no longer be visualized on discrete areas of the nuclear membrane but rather was homogenously distributed over the entire nuclear membrane (Fig. 6 B). A similar effect was previously seen for the ERMES complex, where deleting one subunit resulted in the redistribution of other subunits to the entire organelle (Kornmann et al., 2009). Moreover, in this background, mitochondrial clustering was completely lost, supporting the notion that Tom70 could be a partner protein for Cnm1 on the outer mitochondrial membrane.

Tom70 is a mitochondrial protein import receptor loosely associated with the TOM complex (Dekker et al., 1998). Hence, Tom70 could be affecting Cnm1 indirectly by simply altering the abundance of another mitochondrial OM protein. To uncover if the effect was direct, we first assayed whether Tom70 and Cnm1 interact with one another by performing a coimmunoprecipitation experiment. Indeed, when we pulled down Cnm1, we found a twofold enrichment of Tom70 relative to another TOM component, Tom20 (Fig. 6 C).

To further back-up that the two proteins interact on the opposing membranes, we overexpressed a soluble GFP-Tom70 from its endogenous locus (no full length Tom70 was present in the cells) by deleting its membrane-spanning region (first 38 aa, Δtmd; Wu and Sha, 2006; Brix et al., 2000). While Δtmd GFP-Tom70 is distributed homogenously in the cytosol, overexpression of Cnm1 caused the redistribution of Δtmd GFP-Tom70 to surround the nuclear membrane (Fig. 6 D). High-resolution images also demonstrate that the soluble GFP-Tom70 concentrated around the nuclear membrane in areas that were completely overlapping with the Cnm1-mCherry signal (Fig. 6 E). Moreover, despite the high expression of the cytosolic domain of Tom70 in these cells, there was a complete loss of mitochondrial clustering when Cnm1 was overexpressed (Fig. 6 D).

An additional support for Cnm1 directly binding Tom70 is the presence of an internal mitochondrial targeting signal-like (iMTS-L) sequence in the very C-terminal end of Cnm1 (Schneider et al., 2021; Fig. S5, A and B). Such iMTS-L signals have previously been shown to directly bind Tom70 (Backes et al., 2018). Indeed, a small deletion in this region abrogated the capacity of Cnm1 to increase contact extent (Fig. 6 F) and also resulted in loss of the discrete accumulations of Cnm1 on the nuclear membrane and its homogenous redistribution to the entire nuclear periphery (Fig. 6 F).

To assay the converse interaction, we deleted the predicted membrane-spanning region of Cnm1 (both transmembrane domains [TMDs] in the first 112 aa, Δtmd; Fig. S5 B) and followed its distribution in the cell when tagged with GFP. Indeed, we found that the soluble Cnm1 was no longer on the nuclear periphery but rather was cytosolic, with clear mitochondrial membrane accumulations. These accumulations became even stronger when Tom70 was overexpressed, supporting that recruitment to the mitochondrial OM occurs through Tom70 (Fig. 6 F). What was surprising, however, was that this manipulation of Cnm1 dramatically altered mitochondrial morphology, causing mitochondria to fragment (Fig. S5 C). Since deletion of Cnm1 did not cause this effect, we assume that this is due to buffering of Tom70 binding to its other clients and not loss of Cnm1 activity.

The above experiments highlight the need for Cnm1 to have a Tom70-binding site and for both Cnm1 and Tom70 to be integrated into their respective membranes to enable their tethering function.

## Discussion

Nuclear–mitochondria communication is one of the hallmarks of eukaryotic cells, underlying the tight coordination between energy supply and cellular needs. While mitochondria were already shown to form close proximities with the nucleus in the late 1960s and early 1970s (Baker and Franchi, 1969; Kessel, 1968; Aikawa et al., 1970; Rowley et al., 1971; Franke et al., 1973), the nature and mechanism of these proximities remained unclear. Over the years, several functions were suggested for the interactions between these two organelles, including a role in fission-yeast mitosis (McCully and Robinow, 1971), ATP transfer in cardiac cells (Dzeja et al., 2002), and heme trafficking in *S. cerevisiae* (Martinez-Guzman et al., 2020). However, the difficulties in differentiating the ER from the nuclear envelope have made it challenging to directly identify and study the molecular machinery of the contact.

Here, we present a methodology in yeast to clearly distinguish the contact site between the nucleus and mitochondria from the one formed with the ER. We identify the previously uncharacterized Ybr063c/Cnm1 as a new nuclear membrane protein that acts as a specific tether for mitochondria. Together with Tom70 on the mitochondrial membrane, Cnm1 can function to recruit mitochondria specifically to the nuclear ER. We show that Cnm1 levels are regulated by PC, coupling phospholipid biosynthesis with the extent of contact site formation (Fig. 6 H).

For years, it was assumed that every two organelles can form a singular type of contact between them. However, recent evidence suggests that between two organelles, several distinct contact sites can form, each with specific machinery and function. For example, it was recently shown that between mitochondria and the vacuole in yeast, there are two types of contacts: one mediated by Vam6-Tom40, which has a role in the cellular stress response, and the other mediated by Mcp1-Vps13 and has functions that can bypass the loss of the ERMES complex (González Montoro et al., 2018). The two types of vacuole and mitochondria patches are found adjacent to one another, suggesting that both functions are spatially restricted. Similarly, in mammalian cells, several types of ER–plasma membrane contacts have been identified (Besprozvannaya et al., 2018). Our work extends these findings to the contact between mitochondria and the ER. The discovery that Cnm1, a nuclear envelope protein, mediates nuclear ER-specific contacts that are distinct from ERMES-mediated contacts opens a new molecular window to exploring the intricate structure of nuclear envelope/ER and mitochondrial contact sites.

Why would the ER and mitochondria need to maintain two distinct contact sites? The ERMES-mediated ER–mitochondria contact site is known to have a role in metabolism of phospholipids. It was recently shown that Mmm1 and Mdm12 form a heterocomplex, which can mediate the transfer of phospholipids in vitro. Furthermore, mutations in Mmm1 or Mdm12 resulted in impaired phospholipid transfer in vivo (Kawano et al., 2018). Several observations support the idea that PE transport can occur through ERMES (Kundu and Pasrija, 2020); however, in vitro studies showed that PS to PE conversion rate was reduced in ERMES mutants, suggesting that ERMES mediates the transfer of PS from the ER to mitochondria (Kojima et al., 2016). All of these data suggest a role for the ERMES complex in the initial steps of PC production: the conversion of PS to PE to PC. However, once PC is formed in the nuclear envelope/ER membrane, how does it return to mitochondria, where it makes up more than 40% of its membranes (Sperka-Gottlieb et al., 1988)? We have shown that Cnm1 is regulated by PC levels. In strains deleted for enzymes required for the biosynthesis of PC, levels of Cnm1 are reduced; however, upon the addition of choline, which allows that rescue of PC levels (Carman and Han, 2011), Cnm1 levels are restored. This extends the nucleus–mitochondria contact. It is highly appealing to hypothesize that regulation of Cnm1 levels by PC reflects a role of the nucleus–mitochondrial contact in the transfer of PC from the nuclear envelope/ER, where it is formed, to mitochondria, where it is highly abundant. Interestingly, several PC biosynthesis–related proteins, including the rate-limiting enzyme of the Kennedy pathway Pct1 and the transcriptional regulator Opi1, are enriched in the nuclear ER (Breker et al., 2014; Dubreuil et al., 2019). This suggests that high PC levels may be found specifically in the perinuclear area or that nuclear PC has a regulatory role. However, whether nucleus–mitochondria contacts have indeed a role in the PC transfer to mitochondria remains to be studied. Having the molecular machinery at hand should now make this feasible.

How PC abundance affects Cnm1 levels is still unclear. In our study, Cnm1 was expressed from a constitutive promoter, suggesting that the difference in the levels of Cnm1, observed upon deletion of PC enzymes, is a result of a posttranslational regulatory event. Indeed, other pathways constituents, such as the choline transporter Hnm1, are posttranslationally regulated through phosphorylation and ubiquitination (Fernández-Murray et al., 2013).

An interesting feature of Cnm1 function is that it pairs with Tom70 on the mitochondrial membrane to form the nucleus–mitochondria contact. Tom70 has a well-known role in protein translocation (Dekker et al., 1998) as well as a role in the tubular ER–mitochondria contact site through interactions with Lam6 (Elbaz-Alon et al., 2015; Murley et al., 2015). What would be the cellular benefits of pairing Cnm1 with Tom70, a protein that is already involved in many other interactions? In recent years, several proteins that have roles in protein translocation across organelle membranes were shown to have an additional function as contact site tethers. The ERMES subunit Mdm10 (Kornmann et al., 2009) is also part of the mitochondrial sorting and assembly machinery (SAM; Ellenrieder et al., 2016). The ER–mitochondria contact site proteins Lam6 and ER membrane protein complex (EMC) were shown to interact with two subunits of the TOM translocon, Tom70 and Tom5, respectively (Murley et al., 2015; Elbaz-Alon et al., 2015; Lahiri et al., 2014). Finally, the vacuole–mitochondria tether Vam6 was shown to interact with Tom40 (González Montoro et al., 2018). Having a limiting amount of proteins that can be used either for translocation or for contact site formation might therefore be a general mechanism to balance between lipid and protein abundance in an organelle.

More broadly, our work opens up a new molecular window into an underexplored contact site in yeast. Cnm1, Tom70, as well as the many other proteins that were highlighted by our screen, can serve as tools to now manipulate the extent of the contact and study its various potential functions. A better understanding of how two information hubs, the nucleus and mitochondria, communicate in healthy cells, should provide us with insights into communication failures in disease.

## Materials and methods

### Yeast strains and plasmids

*S. cerevisiae* strains were based on the laboratory strain BY4741 (Brachmann et al., 1998) or SEY6210.1 (Robinson et al., 1988). Genetic manipulations were performed using the lithium acetate, polyethylene glycol, single-stranded DNA method (Gietz and Woods, 2006). Plasmids for PCR-mediated homologous recombination were previously described (Janke et al., 2004; Longtine et al., 1998), and primers were designed using Primers-4-Yeast (Yofe and Schuldiner, 2014). Table S3 and Table S4 list the plasmids and strains used in this study, respectively. Plasmid pRs316-PGK-CFP-HDEL-URA3 (a blue fluorescent protein [BFP] fused to an ER retrieval sequence) was kindly provided by Prof. J. Goodman (University of Texas Southwestern Medical Center, Dallas, TX). The pESC-NLS-TFP plasmid expressing the nuclear marker (tdTomato conjugated to a nuclear localization signal [NLS]) was kindly provided by Prof. D. Kaganovich (Göttingen University, Göttingen, Germany). The plasmid of pADHpr mtBFP426 (BFP fused to a mitochondrial targeting sequence) was kindly provided by Prof. C. Ungermann (Osnabrück University, Osnabrück, Germany). pBS35 mCherry-HygroR plasmid (PCR-mediated homologous recombination for C-terminal tagging with mCherry and hygromycin resistance) was kindly provided by Prof. N. Barkai (Weizmann Institute of Science, Rehovot, Israel). pFA6a-His3MX6-GAL1pr plasmid (PCR-mediated homologous recombination for changing a promoter sequence of a gene using the galactose [GAL] promoter with Nourseothricin [NAT] resistance) was kindly provided by Prof. J. Gerst (Weizmann Institute of Science, Rehovot, Israel).

### Culturing of yeast

Yeast cells were cultured overnight at 30°C in synthetic minimal medium (0.67% wt/vol yeast nitrogen base with ammonium sulfate and amino acid supplements) with glucose (2%; SD) or galactose (2%; SGal). The next day, cells were either diluted and grown until reaching mid-logarithmic phase (0.4–0.9 OD_600_) or kept undiluted for experiments performed in stationary phase (1 < OD_600_).

### Manual fluorescence microscopy and organelle staining

Glass-bottom, 384-well microscopy plates (Matrical Bioscience) coated with Concanavalin A (Sigma-Aldrich) were used for imaging. Cells in stationary or mid-logarithmic phase were adhered to the plates by incubating at RT for 15 min and were then washed and imaged in synthetic minimal medium.

For red mitochondrial staining, upon adherence to the plate, media was replaced with media containing 50 nM MitoTracker (MitoTracker Orange CMTMRos; Invitrogen), and cells were incubated at RT for 10 min, washed once, and imaged. For blue mitochondrial staining, upon adherence to the plate, media was replaced with media containing 500 nM MitoView 405 (MitoView 405; Biotium), and cells were incubated at RT for 10 min, washed three times, and imaged in synthetic minimal medium.

Imaging was performed at RT using a VisiScope Confocal Cell Explorer system composed of a Zeiss Yokogawa spinning disk scanning unit (CSU-W1) coupled with an inverted IX83 microscope (Olympus). Single-focal-plane and Z-stack images were acquired with a 60× oil lens (NA 1.4) and were captured using a PCO-Edge sCMOS camera, controlled by VisiView software (GFP [488 nm], RFP [561 nm], or BFP [405 nm]). Manual inspection and brightness adjustment were performed using ImageJ (Schindelin et al., 2012). Overlap analysis for quantification in Fig. 3 was done by the Artificial Intelligence feature of the ScanR Olympus soft imaging solutions version 3.2.

High-resolution imaging was performed at RT using automated inverted fluorescence microscope system (Olympus) harboring a spinning disk high-resolution module (Yokogawa CSU-W1 SoRa confocal scanner with double micro lenses and 50-µm pinholes). Images of cells in the 384-well plates were using a 60× oil lens (NA 1.42) and with a Hamamatsu ORCA-Flash 4.0 camera. Fluorophores were excited by a laser and images were captured in three channels: GFP (excitation wavelength 488 nm, emission filter 525/50 nm), mCherry (excitation wavelength 561 nm, emission filter 617/73 nm) and DAPI (excitation wavelength 405 nm, emission filter 447/60). All images were taken in a Z-stack, and using cellSens software. Best focal plane for presentation, images were deconvoluted using cellSens software.

### Library preparation and high-throughput screening

The synthetic genetic array method was used for integrating the desired genomic manipulations into yeast libraries (Tong and Boone, 2006; Cohen and Schuldiner, 2011). Query strains for screens were constructed on an synthetic genetic array–ready strain (YMS721; Breslow et al., 2008), and libraries were handled using a RoToR bench-top colony array instrument (Singer Instruments). Briefly, query strains were mated with strains from the library on rich medium plates to generate diploid cells. Cells were then transferred to nitrogen starvation media for 7 d to induce sporulation. Haploid cells were selected using canavanine and thialysine (Sigma-Aldrich) lacking leucine to select for MATalpha. The final library was generated by selecting for the combination of manipulations desired. Representative strains from the final library were validated by both microscopy and check-PCR.

For screens described in Fig. 2, screening was performed using an automated, inverted fluorescence microscopic ScanR Olympus soft imaging solutions system (Breker et al., 2013). Images were acquired using a 60× air lens (NA 0.9, GFP [490 nm], and RFP [572 nm]). For the screen described in Fig. 4, libraries were imaged using a Hamamatsu flash orca 4.0 camera and a CSU-W1 Confocal Scanner Unit of Yokogawa with a 50 µm pinhole disk. The software used was ScanR Olympus soft imaging solutions acquisition 3.2, and images were acquired using a 60× air lens (NA 0.9, GFP [488 nm], and RFP [561 nm]). For all screens, libraries were imaged at RT, during mid-logarithmic growth. Images were manually inspected using ImageJ software (Schindelin et al., 2012).

### EM

The Tokuyasu method was used for imaging (Tokuyasu, 1973). In brief, samples were fixed in 0.1% glutaraldehyde (EMS) and 4% paraformaldehyde (EMS) in 0.1 M cacodylate buffer (prepared from dimethylarsinic acid sodium salt trihydrate; Sigma-Aldrich) containing 5 mM CaCl_2_ (pH 7.4; Sigma-Aldrich) for 2 h and then washed and embedded in 10% gelatin (EMS) and further fixed for 24 h at 4°C. The samples were then cryoprotected by infiltration with 2.3 M sucrose (J.T. Baker) for 48 h at RT and frozen by plunging into liquid nitrogen. Ultrathin (70–90 nm) frozen sections were obtained with a Leica EM UC7 cryo-ultramicrotome and then transferred to formvar-coated 200-mesh nickel transmission EM grids (EMS). Grids were washed and embedded in 2% methyl cellulose (Sigma-Aldrich) and 0.4% uranyl acetate (EMS). Images were acquired using a Thermo Fisher Scientific Tecnai T12 transmission electron microscope equipped with a bottom mounted TVIPS TemCam-XF416 4k × 4k CMOS camera.

### Cryoelectron tomography

For cell vitrification, cryo-EM grids (R1.2/1.3, Cu 200 mesh grid; Quantifoil MicroTools) were glow-discharged in a plasma cleaner (PDC-3XG; Harrick) to charge the surface of the carbon film. The grids were then mounted onto a Vitrobot Mark IV (FEI), and 3.5 µl cell culture (0.8 OD_600_ in YPD) was deposited on the carbon side of each grid before blotting. Blotting was performed from the back of the grid with filter paper (Whatman Filter Paper 597; Sigma-Aldrich) at a strength setting of 10 for 10 s. The grids were plunged immediately after into liquid ethane cooled by liquid nitrogen and quickly transferred to a storage Cryo-box. Cryo-boxes were stored in liquid nitrogen until needed.

For cryo-focused ion beam milling, the frozen grids were mounted into Autogrid carriers (FEI) and secured to them with a copper clip ring. The grids were then inserted in a Scios 2 DualBeam microscope (FEI) under high vacuum and kept at −180°C. The sample was coated with a thin layer of organometallic platinum using a gas injection system to protect it from unnecessary damage from the focused ion beam.

As many as six clusters of <10 cells were selected as milling positions in each grid. The milling process was done with the Ga^2+^ ion beam at an inclination of 20° and in sequential steps, from 30 kV and 500 pA for the elimination of most of the material above and below the plane of interest to 30 kV and 30 pA for the final thinning down. The milling progress was monitored by scanning EM imaging at 3 kV and 8.9 pA, and the resulting lamellas were ∼14 µm wide and 150–200 nm thick. The grids were afterwards stored in Cryo-boxes submerged in liquid nitrogen.

For cryoelectron tomography, the grids were loaded into a Polara cryoelectron microscope (FEI) and kept under high vacuum at −180°C. The microscope was equipped with a 300-kV field emission gun, energy filter (Gatan), and K2 summit direct electron detector (Gatan). Overview pictures of the lamellas were taken at low magnification (4,500×, 27 Å/pixel, and −105 µm defocus) to identify the location of nucleus–mitochondria contact sites. Tilt series were taken at these regions of interest with a unidirectional scheme from −54° to 45° in 3° steps at high magnification (34,000×, 3.509 Å/pixel, and −5 µm defocus) using SerialEM software (https://bio3d.colorado.edu/SerialEM/; RRID SCR_017293; Mastronarde, 2005). The tilt series images were taken in dose-fractionation mode and constant exposure to obtain a final electron dose of ∼120e-/Å2 per tilt series.

For tomogram reconstruction, the different frames that compose each tilt were aligned using TOMOMAN software (https://github.com/williamnwan/TOMOMAN; Nickell et al., 2005), and the resulting aligned images were used to create new tilt series. These new tilt series were aligned in IMOD software (https://bio3d.colorado.edu/imod/; RRID SCR_003297; Kremer et al., 1996) using patch tracking, and the tomograms were reconstructed using back-projection. The tomograms were binned to a voxel size of 14.036 Å for better visualization.

For postprocessing, a deconvolution filter (https://github.com/dtegunov/tom_deconv) was used to improve contrast in the tomograms.

Computational measurements of contact site extent and 3D segmentation analysis of the tomograms were performed as previously described (Salfer et al., 2020).

### Western blot

Four OD_600_ of cells expressing Cnm1 tagged with GFP on a control strain or on the background of *Δcho2*, *Δopi3*, or* Δino2*, with or without 5 mM choline supplementation, were grown in SD complete media until reaching mid-logarithmic phase. Cells were then collected by centrifugation at 3,000*g* for 3 min, subsequently transferred to a fresh 1.5-ml microcentrifuge tube, and washed with 1 ml nuclease-free water. Cells were resuspended in 200 µl lysis buffer (8 M urea, 50 mM Tris, pH 7.5, and protease inhibitors; Merck) and subsequently lysed by vortexing at high speed with glass beads (Scientific Industries) at 4°C for 10 min. 25 µl of 20% SDS was added to each sample before incubation at 45°C for 15 min. The bottom of the microcentrifuge tubes was then pierced, loaded into 5-ml tubes, and centrifuged at 4,000*g* for 10 min to separate the lysate from the glass beads. The flow-through collected in the 5-ml tubes was transferred to a fresh 1.5-ml microcentrifuge tube and centrifuged at 20,000*g* for 5 min. The supernatant was collected and 4x SDS-free sample buffer (0.25 M Tris, pH 6.8, 15% glycerol, and 16% Orange G containing 100 mM DTT) was added to the lysates, which were incubated at 45°C for 15 min.

Protein samples were separated by SDS-PAGE using a 4–20% gradient gel (Bio-Rad) and then transferred onto 0.45-µm nitrocellulose membrane (Pall Corporation) using the Trans-Blot Turbo transfer system (Bio-Rad). Membranes were blocked in SEA BLOCK buffer (Thermo Scientific; diluted 1:5 in PBS) for 1 h at RT and subsequently incubated overnight at 4°C with primary antibodies diluted in a 2% wt/vol BSA/PBS solution containing 0.01% NaN_3_. Primary antibodies used were rabbit anti-GFP (ab290, 1:3,000; Abcam) and rabbit anti-Histone H3 (ab1791, 1:5,000; Abcam). After washing, membranes were then probed with secondary antibody (800CW Goat anti-Rabbit IgG, ab216773; Abcam) diluted 1:10,000 in 5% wt/vol nonfat milk/Tris-buffered saline with 0.05% Tween 20 (TBST) for 1 h at RT. Blots were washed and imaged on the LI-COR Odyssey Infrared Scanner.

### Coimmunoprecipitation

Yeast overexpressing Cnm1-GFP with either Tom20-mCherry or Tom70-mCherry were grown to mid-logarithmic phase, and a total of five OD_600_ were collected by centrifugation and washed once in water. The cell pellets were subsequently resuspended in 500 µl ice-cold lysis buffer (1% digitonin, 150 mM NaCl, 50 mM Tris-HCl, pH 8.0, 5% glycerol, 1 mM MgCl_2_, and protease inhibitors; Merck) and transferred to FastPrep tubes containing 1-mm silica spheres (lysing matrix C; MP Biomedicals). The tubes were loaded into a FastPrep24 instrument (MP Biomedicals), and the cells were lysed by six cycles of 1 min beating at maximum speed, followed by 5 min on ice. Lysates were then centrifuged at 16,000*g* for 10 min at 4°C, and of the 400 µl cleared lysate, 10% was removed as “input,” which was reduced and denatured by incubation at 45°C for 15 min with Laemmli buffer containing 12.5 mM DTT. The rest of the cleared lysate was used for immunoprecipitation by rotation with 30 µl washed GFP-Trap (Chromotek) slurry for 1 h at 4°C. The GFP-Trap beads were subsequently washed three times in 500 µl wash buffer (150 mM NaCl and 50 mM Tris-HCl, pH 8.0), resuspended in 100 µl 2x Laemmli buffer (containing 25 mM DTT), and incubated at 45°C for 15 min before separation by SDS-PAGE. 10% input was loaded relative to the immunoprecipitation samples. Densitometry was performed on Image Studio Lite (LI-COR) software and used to calculate enrichment values.

### Procedures for carbonate extraction (CE)

Isolation of mitochondria from yeast cells was performed by differential centrifugation, as previously described (Daum et al., 1982). On these purified mitochondria, CE was performed. 100 µg mitochondria was purified from a strain overexpressing 3HA-Ybr063c from its genomic locus and resuspended in 100 µl of 200 mM sodium carbonate (Na_2_CO_3_), followed by 30 min incubation at 4°C. Supernatant and pellet fractions representing soluble and membrane-embedded proteins, respectively, were obtained by centrifugation (80,000*g*, 30 min, 4°C). Proteins from the supernatant were extracted by TCA precipitation. TCA was added to final concentration of 12% (wt/vol), and the mixture was incubated for 30 min at 4°C followed by centrifugation (36,800*g*, 15 min, 2°C). The pellet was washed with 100 µl 90% acetone. The mixture was centrifuged again (36,700*g*, 5 min, 2°C), and the pellet containing the proteins was dried at 40°C before analysis. For analysis, both fractions were resuspended in 40 µl of 2x Laemmli buffer, heated for 10 min at 95°C, and analyzed by SDS-PAGE and immunoblotting. Protein samples for immune decoration were analyzed on 12.5% SDS-PAGE and subsequently transferred onto nitrocellulose membranes by semi-dry Western blotting. Proteins were detected by blocking the membrane with 5% milk and subsequently incubating them with primary antibodies (either polyclonal rat anti-HA diluted 1:1,000, polyclonal rabbit anti-Tom20 diluted 1:5,000, or polyclonal rabbit anti-Hep1 diluted 1:3,000) and then with horseradish peroxidase conjugates of goat anti-rabbit secondary antibody.

### Spot assay

Serial dilutions were grown on synthetic minimal medium with either glucose or galactose supplementation. Cells were grown overnight in 2% galactose media containing their respective selections. They were back diluted to an OD_600_ = 0.2 in 2% galactose media and incubated for ∼6 h at 30°C. After at least one cell division or after reaching mid-logarithmic phase, strains were back diluted again to OD_600_ = 0.1 and then diluted in 10-fold increments. Next, 2.5 µl of each dilution was plated using a multichannel pipette (Gilson) on SD and SGal agar plates, both containing all amino acids. Plates were imaged using Canon PC1591 digital camera after 3 d of growth at 30°C.

### Growth assay

The growth assays were performed using a Spark (Tecan) plate reader. Transparent 96-well plates (Greiner) were used. Cells were grown in an incubator (Liconic) at 30°C and shaking at 500 rpm. Samples were measured every 30 min following a strong resuspension on a plate shaker (bioshake 3000) at 1,200 rpm. OD was measured at 600 nm wavelength.

### PC supplementation

PC supplementation was performed as previously described (Grant et al., 2001), with some modifications. Cells were grown to a logarithmic phase in synthetic minimal medium at 30°C and then transferred to 4°C for 15 min. 1 mM of 1-myristoyl-2-{6-[(7-nitro-2-1,3-benzoxadiazol-4-yl)amino]hexanoyl}-*sn*-glycero-3-phosphocholine (NBD-PC; Sigma-Aldrich) diluted in DMSO was added to the cells while the plate was on ice, and after 15 min, cells were imaged as described above.

### Statistical analysis

Statistical analysis was done using two-tailed Student’s *t* tests. Bars represent standard deviation. Data distribution was assumed to be normal, but this was not formally tested.

### Online supplemental material

Fig. S1 shows overexpression of the ERMES complex subunit Mdm34 does not extend the nucleus–mitochondria contact. Fig. S2 shows that Ybr063c (Cnm1) does not affect the ERMES complex and is only partially colocalized with its subunits. Fig. S3 shows that mitochondrial clustering around the nucleus mediated by overexpressing Cnm1 is ERMES independent. Fig. S4 shows that choline addition rescues Cnm1 levels in cells harboring mutation in PC biosynthesis–related genes. Fig. S5 shows that domain architecture of Cnm1 and the effect of losing its TMD on mitochondrial morphology. Table S1 lists all mCherry-tagged proteins that fully or partially colocalized with the nucleus–mitochondria contact site reporter from Fig. 2. Table S2 lists all genes whose deletions altered Cnm1-mediated clustering of mitochondria around the nucleus from Fig. 4. Table S3 lists the plasmids used in this study. Table S4 lists the yeast strains used in this study. Video 1 shows time-lapse imaging of the effect of activating Ybr063c expression from a *GAL* promoter.

## Supplementary Material

Table S1 lists all mCherry-tagged proteins that fully or partially colocalized with the nucleus–mitochondria contact site reporter from Fig. 2.

Table S2 lists all genes whose deletions altered Cnm1-mediated clustering of mitochondria around the nucleus from Fig. 4.

Table S3 lists the plasmids used in this study.

Table S4 lists the yeast strains used in this study.
